# Alterations in Human Milk Adiponectin, Leptin, Resistin, and microRNAs in Mothers With Gestational Diabetes Mellitus Compared With Healthy Controls: A Systematic Review and Network‐Based Analysis

**DOI:** 10.1002/fsn3.72256

**Published:** 2026-08-31

**Authors:** Zhijun Zhang, Maryam Davoudi, Amirreza Ghafourian, Parmida Dehghan, Mojde Ahmadi, Seyed Mohammad Ayyoubzadeh, Xiaolei Miao, Hamid Choobineh, Reza Afrisham

**Affiliations:** ^1^ Hubei Key Laboratory of Diabetes and Angiopathy, School of Pharmacy Xianning Medical College, Hubei University of Science and Technology Xianning Hubei Province P. R. China; ^2^ Department of Medical Laboratory Sciences, School of Allied Medical Sciences Tehran University of Medical Sciences Tehran Iran; ^3^ Students’ Scientific Research Center Tehran University of Medical Sciences Tehran Iran; ^4^ Department of Medical Education, School of Medicine Tehran University of Medical Sciences Tehran Iran

**Keywords:** body weight, gestational diabetes, hormones, human milk, infant obesity

## Abstract

Gestational diabetes mellitus (GDM) is linked to poor infant metabolic outcomes, potentially via altered human milk (HM) hormones and microRNAs. Since their specific roles remain unclear, this study synthesizes current evidence on HM adipose tissue‐derived hormones (particularly adiponectin, leptin, and resistin) and microRNA modifications during gestational diabetes. Firstly, a systematic review following PRISMA 2020 guidelines was conducted (PROSPERO: CRD42024612813). Searches in major databases identified studies comparing HM adiponectin, leptin, or resistin concentrations and/or miRNA profiles between GDM and normoglycemic mothers. Secondly, bioinformatics analysis using miRWalk 3.0, functional enrichment, and network topology mapping examined miRNA interactions with *ADIPOQ, LEP*, and *RETN* genes. Twelve studies were included. Adiponectin showed the most consistent GDM‐associated reductions, though findings were context‐dependent. Leptin was primarily associated with maternal adiposity rather than GDM status. Resistin evidence was insufficient. Three miRNA studies revealed stage‐dependent dysregulation in GDM, with *miR‐148a, miR‐30b, let‐7a*, and *let‐7d* linked to infant growth outcomes during the first 6 months. Bioinformatics identified *miR‐148a‐5p* and *miR‐30b‐3p* as targeting all three adipokine genes, with enrichment in glucose homeostasis and insulin resistance pathways. Network analysis highlighted *TCF7L2, INSR, GCK*, and *HNF1A* as central nodes. GDM is associated with selective alterations in HM adipokines and miRNAs, with adiponectin and specific miRNAs showing the strongest signals. These findings support a conceptual model where GDM shapes HM's molecular composition through interacting endocrine and posttranscriptional mechanisms, potentially influencing infant metabolic programming. Larger longitudinal studies are needed to validate these observations and determine clinical relevance.

## Graphical abstract

Gestational diabetes alters the adipose–mammary gland axis, changing human milk composition—notably reducing adiponectin and dysregulating *miR‐148a, miR‐30b, let‐7a*, and *let‐7d*, which target *ADIPOQ*, *LEP*, and *RETN* and are enriched in glucose homeostasis/insulin resistance pathways. Via breastfeeding, this altered milk miRNA profile may shape infant growth outcomes (body fat, length, weight, WAZ, WLZ) over the first 6 months. Abbreviations: miR, miRNA; WAZ, Weight‐for‐age Z‐score; WLZ, Weight‐for‐length Z‐score.

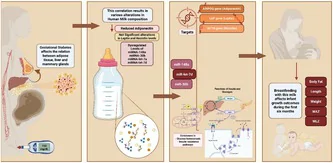

**Abbreviations**
3′‐UTR: 3′ untranslated regions
AMPK: AMP‐activated protein kinase
APJ: Apelin receptor
BMI: body mass index
CRP: C‐reactive protein
GDM: gestational diabetes mellitus
HM: human milk
HMW: high molecular weight
MFE: minimum free energy
miRNAs: microRNAs
MREs: miRNA response elements per target
MUFA: monounsaturated fatty acids
PPARα: peroxisome proliferator‐activated receptor alpha
sEVs: small extracellular vesicles
SFA: saturated fatty acids
SGA: small for gestational age
WFL: weight‐for‐length

## 1 Introduction

Overweight and obesity in children have become a major global health concern, with prevalence rising sharply over the past decades. Globally, more than 340 million children and adolescents aged 5–19 years were classified as overweight or obese in 2016 alone, representing a substantial increase compared with previous years (Zehravi et al. 2021; Murray et al. 2020). A major contributor to this burden is maternal metabolic health during pregnancy, as maternal obesity has been associated with more than a twofold increase in the risk of childhood obesity (Isganaitis et al. 2019). These adverse metabolic trajectories often persist into adulthood, increasing the likelihood of hypertension, Type 2 diabetes (T2D), and coronary heart disease (Van Syoc et al. 2024). Among the maternal conditions implicated in these outcomes, gestational diabetes mellitus (GDM) is particularly important because of its well‐documented adverse effects on both maternal and infant health (Farahvar et al. 2019; Ornoy et al. 2021; Nijs and Benhalima 2020; Pathirana et al. 2020; Kong et al. 2020).

GDM is defined as glucose intolerance first detected during pregnancy and affects a substantial proportion of births worldwide. The International Diabetes Federation estimates that maternal hyperglycemia affects approximately 21.3 million infants annually, with GDM accounting for the majority of these cases (Amirian et al. 2020). This metabolic disorder is characterized by impaired early‐phase insulin secretion, inappropriate beta‐cell function, and consequent fasting and postprandial hyperglycemia (Ornoy et al. 2021; Barbour 2019; Karagoz et al. 2022). These abnormalities contribute to maternal hyperglycemia and dyslipidemia, which can disrupt normal fetal development (Karagoz et al. 2022; Kc et al. 2015; Skrypnik et al. 2019). Elevated maternal glucose levels enhance transplacental nutrient transfer, promoting fetal hyperinsulinemia, excessive fat deposition, and macrosomia, often accompanied by increased body fat and altered anthropometric measures (Ornoy et al. 2021; Amirian et al. 2020; Barbour 2019; Kc et al. 2015). Beyond the neonatal period, children exposed to GDM in utero show an increased risk of overweight and obesity later in life, with risk estimates rising further with age (Gao et al. 2022). Maternal obesity may further intensify these risks, highlighting the combined influence of maternal adiposity and GDM on offspring metabolic outcomes (Weir et al. 2024).

Breastfeeding is widely recognized as a protective factor against childhood obesity and related metabolic disorders. In infants born to mothers with GDM, higher breastfeeding intensity during the first year of life has been associated with slower postnatal weight gain and healthier growth trajectories (Gunderson et al. 2018). Human milk (HM) is not merely a source of nutrients; it is a dynamic biofluid that contains bioactive molecules involved in metabolic regulation and developmental programming. Among these are adipokines, including adiponectin, leptin, and resistin, which are secreted by adipose tissue and have important endocrine roles in energy homeostasis, insulin sensitivity, inflammation, and glucose metabolism. These adipokines are biologically relevant because they reflect maternal metabolic status and may influence infant metabolic development through exposure during early life (Lis‐Kuberka et al. 2024; Galante et al. 2020, 2021; Choi et al. 2022; Nunes et al. 2017; Yu et al. 2018; Balcells‐Esponera et al. 2024; Jin et al. 2026; Bruder et al. 2026). Consistent with this concept, studies in obesity and T2D have shown that these adipokines are altered in metabolic disease states (Ayman et al. 2019), supporting their selection as key targets for our investigation. Their selection is further supported by evidence showing that obese and obese diabetic individuals have significantly higher serum leptin and resistin levels than healthy controls, whereas adiponectin concentrations are significantly lower in obese and obese diabetic subjects. Notably, weight loss has been associated with significant improvement in all three parameters, highlighting their responsiveness to metabolic status (Lis‐Kuberka et al. 2024; Galante et al. 2020, 2021; Choi et al. 2022; Nunes et al. 2017; Yu et al. 2018; Balcells‐Esponera et al. 2024; Jin et al. 2026; Bruder et al. 2026; Ayman et al. 2019).

In addition to its role in metabolic hormones, HM is an excellent source of noncoding RNAs, most notably microRNAs (miRNAs). miRNAs are now acknowledged as integral to postnatal epigenetic regulation (Van Syoc et al. 2024). Binding to the 3′ untranslated regions (3′‐UTR) of messenger RNAs, miRNAs regulate aspects of cell growth, insulin signaling, and glucose metabolism pathways (Shah et al. 2021; Saliminejad et al. 2019; Mirra et al. 2018). In HM, miRNAs are packaged within exosomes, small extracellular vesicles (sEVs) that protect them from enzymatic degradation, allowing them to remain biologically active (Alsaweed et al. 2016). The composition of milk miRNAs changes across lactation stages, from colostrum (days 1–5) through transitional milk (days 6–14) to mature milk (week 2 onward), and is further altered in mothers with GDM (Zheng et al. 2023; Hicks et al. 2022; Shah et al. 2022a). Although milk miRNA profiles change across lactation and may differ in GDM, the available evidence is fragmented. Moreover, only one study has reported a direct association between HM miRNAs and metabolic hormones, indicating a major gap in integrated analyses of these molecular pathways (Zamanillo et al. 2019).

Therefore, this study was designed to address these gaps through a dual‐method framework combining systematic review and bioinformatics analysis. The objective was to synthesize the available evidence on HM alterations in GDM with specific emphasis on the three major adipokines, i.e., adiponectin, leptin, and resistin, evaluate their associations with infant outcomes, and explore predicted miRNA‐hormone gene networks and enriched biological pathways relevant to metabolic regulation. By integrating published clinical findings with computational target analysis, this work aimed to provide a more focused understanding of the molecular signatures of GDM in HM and their potential relevance to early‐life metabolic programming.

## 2 Methodology

### 2.1 Systematic Review

#### 2.1.1 Search Strategy

This systematic review was conducted in accordance with the Preferred Reporting Items for Systematic Reviews and Meta‐Analyses (PRISMA 2020) guidelines (Page et al. 2021). The review protocol was prospectively registered in PROSPERO (CRD42024612813). The search strategy employed combinations of Medical Subject Headings (MeSH) terms and free‐text keywords, including “Gestational Diabetes Mellitus”, “Gestational Diabetes”, “GDM”, “Human Milk”, “Breast Milk”, “MicroRNA”, “miRNA”, “Adiponectin”, “Leptin”, and “Resistin”. The complete search queries are presented in the Supplementary Files. All retrieved records were imported into EndNote X9 software, and duplicate entries were identified and removed prior to screening.

#### 2.1.2 Eligibility Criteria

The eligibility of identified studies was established using a structured Population, Exposure, Comparator, Outcomes, and Study Design (PECOS) framework. For the population criteria, eligible studies included lactating mothers diagnosed with GDM during pregnancy and their breastfed infants. Human milk samples could include colostrum, transitional milk, or mature milk collected at any stage of lactation. GDM diagnosis had to be confirmed using established clinical criteria, including oral glucose tolerance tests, glucose challenge tests, medical records, hospital charts, or documented physician diagnosis. The comparator was apparently healthy, normoglycemic lactating mothers. The primary outcomes were differences in HM miRNA expression profiles and differences in HM concentrations of the three main fat‐derived metabolic hormones, namely adiponectin, leptin, and resistin, between GDM and control groups. Studies that reported additional HM hormones were also considered eligible, provided that they included assessment of at least one of these three target hormones. Secondary outcomes included associations between HM miRNAs and these metabolic hormones, as well as infant growth‐related parameters, including birth weight, weight gain, length, head circumference, weight‐for‐age z‐scores, length‐for‐age z‐scores, body mass index, or other reported infant anthropometric indices. Studies evaluating either HM miRNAs or the three target metabolic hormones in relation to GDM and infant growth were eligible if they addressed the primary research questions. Eligible study designs included original, peer‐reviewed human studies, such as cohort, longitudinal, prospective, observational, comparative, case–control, cross‐sectional, and randomized controlled studies.

Studies were restricted to full‐text publications in English. Publications were excluded if they were reviews, editorials, commentaries, conference abstracts without complete data, protocols, or studies lacking primary data. Animal studies, in vitro experimental studies without HM samples, and studies assessing non‐HM were also excluded. Studies evaluating maternal metabolic disorders other than GDM, such as pre‐existing Type 1 diabetes, T2D, or polycystic ovary syndrome, without a distinct GDM group were excluded as well. In addition, studies assessing maternal serum, maternal plasma, or infant systemic biofluids without direct analysis of HM samples were omitted.

Potential confounding variables, effect modifiers, and secondary exposures, including maternal body mass index, obesity, dietary patterns, ethnicity, mode of delivery, gestational hypertensive disorders, and smoking status, were not used as exclusion criteria, provided that maternal GDM status and the corresponding HM outcomes were clearly reported. Notably, in the present review, HM miRNAs were considered irrespective of their precise cellular origin, since the available studies generally report total milk or exosome‐associated miRNA content without definitively distinguishing between mammary epithelial, immune cell, or other maternal sources. Similarly, the origin of milk‐borne adipokines may involve contributions from the mammary gland, maternal circulation, and adipose tissue. Therefore, the identified miRNA‐hormone associations should be interpreted cautiously as biologically plausible but not mechanistically confirmed relationships.

#### 2.1.3 Study Selection and Data Extraction

The literature screening was conducted in three distinct stages. Initially, all bibliographic citations were imported into EndNote X9 for automated and manual deduplication. Two reviewers (AR.GH. and P.D) independently screened the titles and abstracts of the remaining records using the predefined eligibility criteria. In the second stage, the full‐text articles of all potentially eligible studies were retrieved and assessed independently by the same two reviewers to confirm adherence to the inclusion criteria. Disagreements arising during the selection process were resolved through consensus, and unresolved discrepancies were adjudicated by a third reviewer (M.D).

Data from the included studies were extracted using a predefined data extraction form. Extracted information included study characteristics, such as first author, publication year, study design, sample size, and country or study population; HM characteristics, including concentrations of the primary target hormones (adiponectin, leptin, and resistin) and miRNA expression profiles; infant‐related outcomes, including growth and anthropometric indices. If studies reported additional HM hormones alongside adiponectin, leptin, or resistin, these data were also recorded for contextual interpretation. For all synthesized outcomes, the statistical significance of reported differences, associations, and correlations was extracted and summarized according to the *p*‐values presented in the original publications.

#### 2.1.4 Risk of bias Assessment

The quality of included studies was independently evaluated by three reviewers, with discrepancies resolved by a fourth reviewer. For randomized clinical trials, we used the Cochrane Collaboration Risk of Bias Tool. Newcastle‐Ottawa Scale for cohort and case–control studies. Data is presented in Figure S1.

### 2.2 Bioinformatics Analysis

Computational analyses were performed to identify potential interactions between HM miRNAs and genes encoding the metabolic and peptide hormones. Predicted and validated human miRNA‐mRNA interaction data were obtained from the miRWalk 3.0 database. The downloaded datasets contained specific identifiers, including miRNA ID, target gene symbol, binding probability (binding_p_), minimum free energy (energy), seed‐match status, genomic location (5′ untranslated region, coding sequence, or 3′ untranslated region), experimental validation status, and database support annotations from TargetScan and miRDB.

A predefined target panel consisting of *LEP, ADIPOQ*, and *RETN* genes was selected for evaluation using miRWalk 3.0. For each unique miRNA‐mRNA pair, interaction records at the individual binding site level were aggregated into a composite edge score using a weighted scoring algorithm: “Score = 0.40 × max(bindingp) +0.25 × mean(bindingp) + 0.15 × normalized site count + 0.10 × normalized ∣energy∣ + evidence bonus”.

The evidence bonus component was allocated based on the source of validation and target prediction support, where a bonus of +0.15 was applied for experimentally validated interactions, +0.08 for TargetScan prediction support, +0.05 for miRDB prediction support, and + 0.05 for seed‐match confirmation, with each scoring tier capped. Based on the calculated composite scores, the miRNA‐mRNA pairs were classified into four tiers corresponding to score quartiles: moderate, good, strong, or very strong.

#### 2.2.1 Functional Enrichment and Network Analysis

Functional enrichment analyses were performed using the gseapy wrapper for the Enrichr engine. Enrichment was assessed against the Gene Ontology (GO) Biological Process 2023, GO Molecular Function 2023, Kyoto Encyclopedia of Genes and Genomes (KEGG) 2021 Human, and WikiPathways 2023 Human libraries. The enrichment calculations were applied to two discrete gene datasets, consisting of the full panel of 27 hormone‐related genes and the target genes of eight specific GDM‐ and lactation‐associated miRNAs, including *miR‐148a‐5p, miR‐148a‐3p, miR‐30b‐5p, miR‐30b‐3p, let‐7a‐5p, let‐7a‐3p, let‐7d‐5p*, and *let‐7d‐3p*. Enrichment terms were determined to be statistically significant using a Benjamini‐Hochberg adjusted significance threshold of *p* < 0.05.

To characterize and visualize the topological relationships between miRNAs and target mRNAs, bipartite interaction networks were generated using the NetworkX library. Hub analysis was performed utilizing the top 5000 composite‐scoring miRNA‐mRNA edges derived from the entire panel. Degree centrality metrics were calculated for each node to identify highly connected regulatory miRNAs and target metabolic genes. Separate subnetwork models were constructed to isolate experimentally validated interactions and target interactions associated specifically with the key GDM‐ and lactation‐related miRNAs. All computational steps, data processing, and visualization routines were implemented in Python 3 using the pandas, numpy, matplotlib, seaborn, gseapy, and NetworkX packages.

## 3 Results

### 3.1 Study Selection Data

**FIGURE 1:**
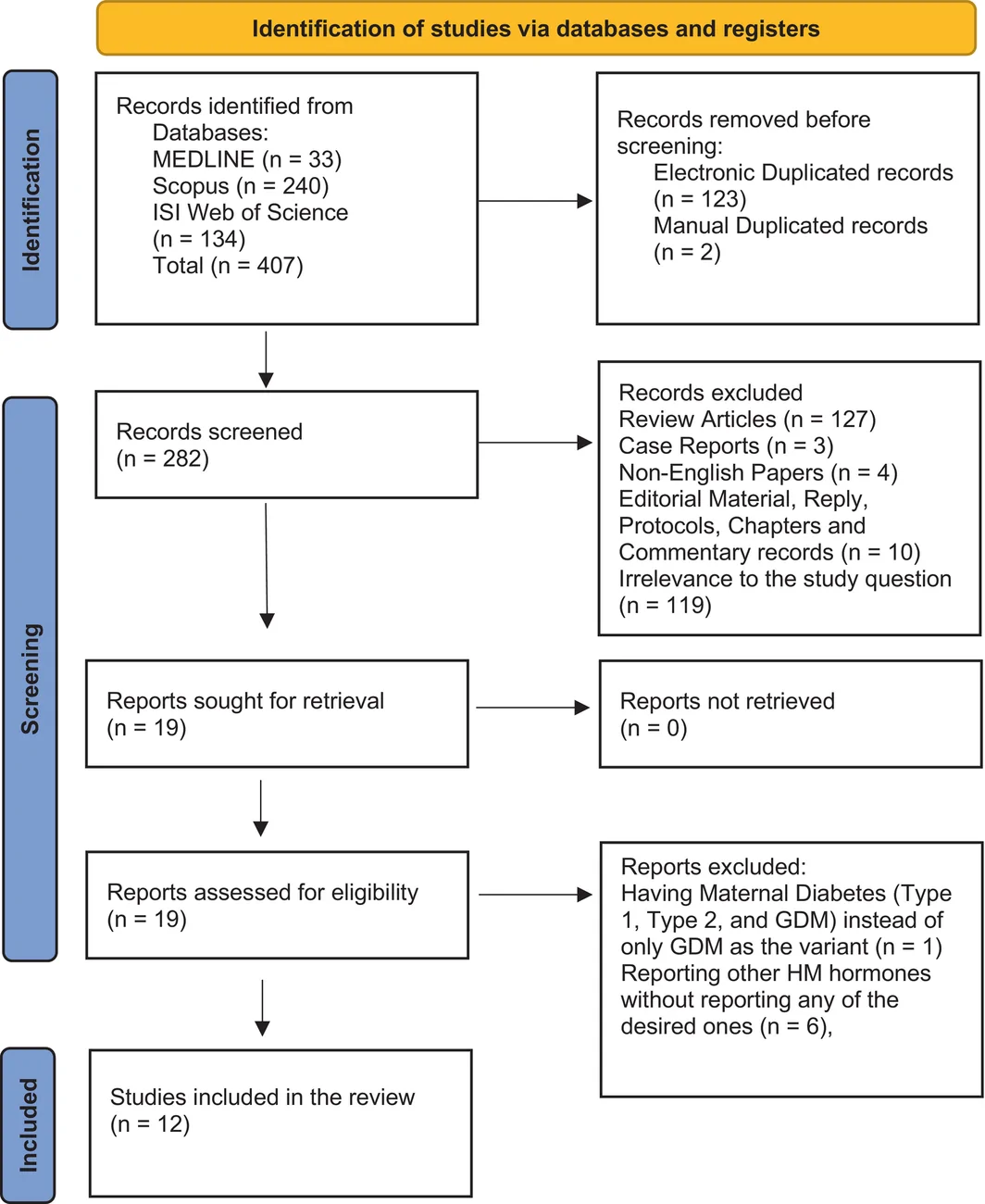
This study's strategy search is shown using the PRISMA flow diagram.

The systematic review process retrieved 407 unique citations from the designated databases. Following the removal of 125 duplicate records, the remaining 282 articles underwent title and abstract screening. Of these, 19 full‐text articles were evaluated against the inclusion and exclusion criteria, leading to the exclusion of seven studies due to incomplete data or failure to meet selection standards. A total of 12 studies met all eligibility criteria and were included in the final qualitative synthesis (Figure 1). The final synthesis comprised nine studies investigating HM metabolic hormones (Lis‐Kuberka et al. 2024; Galante et al. 2020, 2021; Choi et al. 2022; Nunes et al. 2017; Yu et al. 2018; Balcells‐Esponera et al. 2024; Jin et al. 2026; Bruder et al. 2026) and three studies evaluating HM miRNAs (Zheng et al. 2023; Hicks et al. 2022; Shah et al. 2022a). Relevant clinical, demographic, and laboratory parameters extracted from these investigations were organized systematically based on hormonal evaluations (Table 1) and miRNA profiles (Table 2).

**TABLE 1:** Comparison of breast milk hormone levels among breastfeeding mothers with GDM compared to their healthy counterparts.

Study characteristics		Participant characteristics						Findings		
First author (year; country) and study designs	Sample types	GDM and related conditions, GDM diagnosis method; cut‐offs	Number of participants; ethnicity	Body weight or BMI (Mean ± SD)	Age (Mean ± SD)	Weeks after birth	Infant characteristics	Metabolic milk hormones	Changes in hormonal compositions	Other findings
Lis‐Kuberka (2024; Poland) Cohort (Lis‐Kuberka et al. 2024)	Colostrum	An abnormal fasting blood glucose level and/or oral glucose tolerance test (OGTT) following ingestion of 75 g of glucose	Hyperglycemic and GDM: 30; Subgroups: G1: Received postpartum diet treatment (*n* = 16); G2: Received postpartum diet and insulin (*n* = 14); Normoglycemic and non‐GDM: 21 All participants with the ethnicity of White Europeans	GDM‐G1: 22.86 ± 4.12 GDM‐G2: 27.70 ± 5.62 Non‐GDM: 21.90 ± 3.25	GDM‐G1: 32.94 ± 4.68 GDM‐G2: 34.79 ± 5.32 Non‐GDM: 32.43 ± 3.63	1–7 Days of Lactation	All infants had appropriate birth‐weight for gestational age	Ghrelin, Adiponectin, Leptin, Resistin	Lower Colostrum Ghrelin in the G1 group compared to the G2 and non‐GDM (*p* = 0.01), with no difference for leptin, adiponectin, and resistin	Maternal treatment impacts ghrelin levels; Effective management of GDM may result in stable levels of leptin and adiponectin
Galante (2020; Finland) Longitudinal cohort (Galante et al. 2020)	Milk	Diagnosis of GDM based on ICD‐10‐CM Codes O24	501 Mothers, 507 Children with a Total of 501 HM samples for analysis Grouping by mothers BMI GDM present in 44 mothers Ethnicity Not Available	Underweight < 18.5 Normal weight 18.5–24.9 Overweight 25–29.9 Obese > 29.9	31.1 ± 4.2	2.6 ± 0.4 months after birth	Not mentioned	Leptin, Adiponectin, IGF‐1	No significant association between GDM and Adiponectin, Leptin, and IGF‐1 Adiponectin concentrations were observed to be different for male and female infants in relation to GDM (*p* = 0.031)	Higher leptin in milk from overweight and obese mothers (*p* < 0.001) Infant birth weight influenced adiponectin levels in milk (*p* < 0.02)
Galante (2021; Finland) Longitudinal cohort (Galante et al. 2021)	Milk	Clinical information of GDM from hospital records	169 Mothers, 191 infants A total of 352 collected and analyzed samples; GDM present in 36 mothers Ethnicity: Caucasian 38.7% Māori 10.5% Asian 33.5% Pacific Islander 15.7% Other 1.6%	Not mentioned	31.6 ± 5.7	day 5 + 2 day 10 ± 2 4 months±2 weeks	Infant anthropometry at birth (Mean, SD) Weight (g) 2104.3 (416.7) Weight (z‐scores) −0.1 (0.9) Length (cm) 44.5 (3.0) Length (z‐scores) 0.2 (1.1) Head circumference (cm) 31.2 (1.5) Head circumference (z‐scores) 0.3 (0.9)	Leptin, Adiponectin, IGF‐1	Significantly lower HM adiponectin in mothers with GDM (*p* < 0.001)	Significant correlation between Leptin and IGF‐1 concentrations at days 5 and 10 (*p* < 0.05) Association between Maternal ethnicity and differences in HM leptin (*p* = 0.024) and adiponectin (*p* = 0.001) with Māori and Pacific Island groups having lower adiponectin concentrations than Caucasians. Significantly higher HM leptin in singleton vs. multiple pregnancy (*p* = 0.020) Association between C‐Section and lower HM Leptin (*p* = 0.007) and IGF‐1 (*p* = 0.005)
Choi (2022; USA) Prospective cohort (Choi et al. 2022)	Milk	Standard 1‐h, 50‐g glucose challenge test between gestational weeks 26–28 + Serum glucose measurement at 4 times; Cut‐Offs: 5 mg/dL (fasting), 180 mg/dL (1 h), 155 mg/dL (2 h), and 140 mg/dL (3 h)	GDM: 35 Ethnicity: Hispanic or Latino (2.9%) and Not Hispanic or Latino (97.1%) Non‐GDM: 155 Ethnicity: Hispanic or Latino (1.3%) and Not Hispanic or Latino (98.7%)	No‐GDM: 26.5 ± 4.7 GDM: 29.6 ± 7.4	No‐GDM: 39.8 ± 1.1 GDM: 38.2 ± 1.9	4 weeks (±5 days) 12 weeks (±10 days)	Full breastfed (not mentioned more data)	Leptin, Adiponectin, Insulin	Lower milk insulin at 1 month (*p* < 0.03) and 3 months (*p* < 0.003) in the GDM group compared to non‐GDM women; Lower milk glucose at 1 month (*p* < 0.02) and 3 months (*p* < 0.01) in the GDM group compared to non‐GDM women; No significant difference in leptin and adiponectin levels	Higher levels of CRP in the GDM group (*p* < 0.001)
Nunes (2017; Brazil) Observational (Nunes et al. 2017)	Colostrum and mature milk	GDM and hypertension Only mentioned that the conditions were previously diagnosed by prenatal physicians	GDM:25 White: 50% HTN: 23 White: 100% SMS: 58 White: 57.9% SGA: 25 White: 25% CTL: 83 White: 57.1%	GDM: 31.86 ± 6.14 HTN: 32.31 ± 4.06 SMS: 30.24 ± 5.30 SGA 24.66 ± 2.77 CTL 30.95 ± 5.05	GDM: 26.64 ± 5.75 HTN: 27.81 ± 11.22 SMS: 26.28 ± 5.96 SGA: 22.35 ± 5.64 CTL: 27.54 ± 7.49	Colostrum: 24–48 h postpartum, Mature milk: 4 weeks postpartum	SGA infants are predominantly in the SGA group	Leptin, Adiponectin, Insulin	No significant correlation between GDM and the hormone composition of maternal colostrum and mature milk.	A decrease in leptin (*p* = 0.050) and insulin (*p* = 0.012) from colostrum to mature breast milk for the SGA group Correlation between Leptin concentrations in colostrum and mature breast milk with pre‐gestational maternal BMI (*p* < 0.001), as well as at delivery (*p* < 0.001) and at 1 month postnatal (*p* < 0.001)
Yu (2018; China) Longitudinal (Yu et al. 2018)	Colostrum and Mature milk	GDM and BMI; 1‐h 50 g GLT plasma glucose level > 7.8 mmol/L at 24–28 weeks of gestation underwent a 3‐h 100 g OGTT after a 12‐h fast; Diagnose: At least two glucose values met the Carpenter–Coustan criteria (*Diabetes Care* 2014)	GDM: 48 Healthy: 48 Ethnicity Not Available	Pre‐Pregnancy 22.87 ± 3.20 20.71 ± 3.27	GDM: 32.15 ± 3.84 Healthy: 32.04 ± 3.58	Colostrum: 3 days postpartum Mature Milk: 42 and 90 days postpartum	Infant anthropometry at birth (GDM/Healthy) (Mean ± SD) Weight (g) 3468.85 ± 425.87/3342.50 ± 440.36 Height (cm) 50.13 ± 1.59/49.90 ± 1.60 Head circumference (cm) 34.64 ± 1.06 34.30 ± 1.03	Adiponectin, Leptin, Insulin, Ghrelin	Lower concentration of adiponectin and ghrelin in GDM mothers compared to healthy mothers at in colostrum (*p* < 0.001, *p* = 0.011, respectively) and mature milk at day 90 (*p* = 0.009, *p* < 0.001, respectively); Increased insulin in colostrum (*p* = 0.047) and mature milk (*p* = 0.021) of GDM mothers	Adiponectin was inversely associated with infant growth (*p* < 0.001); it was positively correlated with maternal BMI (*p* = 0.001); Leptin and Insulin concentration was positively associated with pre‐pregnancy BMI (*p* = 0.017, *p* < 0.001, respectively) and maternal BMI during lactation (*p* < 0.001 for both) Inverse association of ghrelin concentrations and pre‐pregnancy BMI (*p* = 0.031), and maternal BMI during lactation (*p* < 0.001)
Esponera (2024; Spain) Observational (Balcells‐Esponera et al. 2024)	Milk	Data based on Clinical charts and self‐reported information	Results based on samples of 100 mothers of 120 neonates GDM present in 8 mothers Ethnicity: Caucasian 66% Hispanic 24% Other 10%	Underweight (< 18.5) 10.6% Healthy weight (18.5–24.9) 59.6% Overweight (25–29.9) 16% Obese (≥ 30) 13.8%	32.4 ± 5	4 weeks postpartum	Infant anthropometry at birth (Mean ± SD) Weight (g)/ZS 1161 ± 363/0.25 ± 1.23 Length (cm)/ZS 37.7 ± 3.9/0.09 ± 1.07 Head circumference (cm)/ZS 25.8 ± 2.5/ –0.19 ± 0.97	Adiponectin, Leptin, Insulin, MFG‐E8	Higher Adiponectin in mothers with GDM (*p* = 0.44)	Strong correlation between leptin and pre‐pregnancy BMI (*p* < 0.0001) A greater decrease in length ZS from birth to 28 days in neonates fed milk higher in adiponectin (*p* = 0.003), remaining significant after adjustment for a history of GDM (*p* = 0.026)
Jin (2026; Australia) Cross‐Sectional (Jin et al. 2026)	Milk	Data based on self‐reported information	Low‐Milk Supply (LMS) (*n* = 66) vs. Normal‐Milk Supply (NMS) (*n* = 159) GDM: 37.9% of LMS and 34.0% of NMS	LMS: 27.8 ± 6.8 NMS: 25.7 ± 5.6	LMS: 34.3 ± 4.0 NMS: 33.3 ± 4.4	1–6 months postpartum	Birth weight (g) LMS: 3339 ± 460 NMS: 3421 ± 438 Birth WAZ LMS: 0.1 ± 0.9 NMS: 0.2 ± 0.9	Adiponectin, Leptin, Insulin	None of HM component concentrations were significantly associated with GDM.	Positive correlation between insulin and leptin levels with Maternal Obesity (*p* < 0.001)
Cohort (Bruder et al. 2026)	Milk	Data based on self‐reported information	70 infant‐mother dyads GDM present in 11% Ethnicity: Non‐Hispanic white 74% Non‐Hispanic black 9% Hispanic of any race 1% Non‐Hispanic and another race or multiracial 17%	27.2 ± 5.9	31.5 ± 5	2 months postpartum	Birthweight (kg) (Mean ± SD) 3.43 (0.36)	Leptin Adiponectin	No significant association with GDM	Significant association between HM leptin and pre‐pregnancy BMI (*P* < 0.05)

Abbreviations: CRP, C‐reactive protein; CTL, control; HTN, Gestational Hypertension; HTN, Gestational Hypertension; HTN, Gestational Hypertension; IGF‐1, Insulin‐like growth factor‐1; NGT, normal glucose tolerant; NW, normal weight; OW, overweight; SGA, small‐for‐gestational‐age; SMS, smoking; WAZ, weight‐for‐age Z‐score; ZS, Z‐Score.

**TABLE 2:** Comparison of human breast milk hormone levels among breastfeeding mothers with GDM compared to their healthy counterparts.

Reviewed articles characteristics				Participant characteristics			Results		
First author	Study design	GDM or Its related risk factors	Type of sample	Number of participants; ethnicity	Body weight or BMI	Age (year)	miRNA related to maternal obesity/diet/delivery type and time	miRNAs related to infant obesity	miRNAs related to GDM
Zheng (2023; China) (Zheng et al. 2023)	In vivo and In vitro Studies on Human Milk	GDM(+) vs. GDM(−)	Colostrum (0–7 days postpartum), transitional milk (7–15 days postpartum), and mature milk (after 15 days postpartum)	Mothers with GDM (*n* = 25) Healthy Mothers (*n* = 25) Ethnicity Not Available	The BMI of all participants ranged from 20 to 25 kg/m^2^	20–45	Exosomal miRNA profile of Human milk of GDM mothers had 176 upregulated and 47 downregulated miRNAs compared with the profile in Healthy Mothers (*p* < 0.05)		
Shah (2023; USA) (Shah et al. 2022a)	Cohort	GDM(+) vs. GDM(−)	Milk	Non‐GDM mothers (*n* = 62); 87% Non‐Hispanic White Mothers with GDM (*n* = 32); 69% Non‐Hispanic White	Obese (pre‐pregnancy BMI ≥ 30 kg/m^2^) Overweight (pre‐pregnancy BMI of 25–< 30 kg/m^2^) Normal weight (pre‐pregnancy BMI of 18.5–< 25 kg/m^2^)	21 to 45 years	No significant correlation with Maternal BMI Downregulated by Diet: *miRNA‐148a, miRNA‐30b, miRNA‐let‐7a, miRNA‐let‐7d* (*p* < 0.05)	Negative Correlation at 1 month and again 6 months: *miR‐148a* (*p* < 0.05) Positive Correlation at 1 month: *miRNA‐30b* (*p* < 0.05) Negative Correlation at 3 months: *miRNA‐let‐7a* (*p* = 0.007) Positive Correlation at 3 months: *miRNA‐let‐7d* (*p* = 0.038)	Downregulated in GDM: *miRNA‐148a, miRNA‐30* (*p* < 0.05) *miRNA‐let‐7a, miRNA‐let‐7d* (*p* < 0.01)
Hicks (2022; USA) (Hicks et al. 2022)	Longitudinal Cohort	Maternal BMI and GDM	Milk from 0, 1, and 4 months post‐delivery	192 Mothers 79% White 6% African‐America 6% Asian 4% Biracial 5% Others GDM present in 11%	(Mean ± SD) 27.6 ± 6.6	19–42	*miR‐766‐3p* at 1 month (*p* = 0.042)	No significant correlation with infant obesity	No significant correlation with GDM

### 3.2 HM Metabolic Hormones

Table 1 shows a comparison of milk hormone levels among breastfeeding mothers with GDM compared to healthy counterparts. Nine studies evaluated HM metabolic hormones in mothers with GDM compared with normoglycemic controls, including cohort, longitudinal, prospective, observational, and cross‐sectional designs. Across these studies, milk samples were collected from colostrum to mature milk, spanning the first postpartum days to as late as 6 months of lactation (Lis‐Kuberka et al. 2024; Galante et al. 2020, 2021; Choi et al. 2022; Nunes et al. 2017; Yu et al. 2018; Balcells‐Esponera et al. 2024; Jin et al. 2026; Bruder et al. 2026).

#### 3.2.1 Adiponectin

Among the included hormones, adiponectin was the most commonly reported to differ by maternal GDM status, although results were not fully consistent. Galante et al. (2021) found significantly lower HM adiponectin concentrations in mothers with GDM than in controls (*p* < 0.001) (Galante et al. 2021). Similarly, Yu et al. (2018) reported lower adiponectin concentrations in GDM mothers both in colostrum and in mature milk at day 90 (*p* < 0.001 and *p* = 0.009, respectively) (Yu et al. 2018). In contrast, no significant association between GDM and HM adiponectin was found in (Balcells‐Esponera et al. 2024), Lis‐Kuberka et al. (2024) (Lis‐Kuberka et al. 2024), Galante et al. (2020) (Galante et al. 2020), Choi et al. (2022) (Choi et al. 2022), Nunes et al. (2017) (Nunes et al. 2017), Jin et al. (2026) (Jin et al. 2026), or Bruder et al. (2026) (Bruder et al. 2026) studies.

Several studies also linked adiponectin to infant or maternal characteristics independent of GDM. In Galante et al. (2020), adiponectin concentrations differed by infant sex in relation to GDM (*p* = 0.031), and infant birth weight influenced HM adiponectin levels (*p* < 0.02) (Galante et al. 2020). Yu et al. (2018) found that adiponectin was inversely associated with infant growth (*p* < 0.001) and positively correlated with maternal BMI (*p* = 0.001) (Yu et al. 2018). Likewise, Balcells‐Esponera et al. (2024) reported that higher milk adiponectin was associated with a greater reduction in length z‐score from birth to 28 days (*p* = 0.003), and this association remained significant after adjustment for maternal GDM history (*p* = 0.026) (Balcells‐Esponera et al. 2024). In Galante et al. (2021), maternal ethnicity was also associated with adiponectin differences (*p* = 0.001), with Māori and Pacific Island mothers showing lower concentrations than Caucasian mothers (Galante et al. 2021).

#### 3.2.2 Leptin

In contrast to adiponectin, HM leptin did not appear to differ consistently by GDM status. Lis‐Kuberka et al. (2024) (Lis‐Kuberka et al. 2024), Galante et al. (2020) (Galante et al. 2020), Choi et al. (2022) (Choi et al. 2022), Nunes et al. (2017) (Nunes et al. 2017), Jin et al. (2026) (Jin et al. 2026), and Bruder et al. (2026) (Bruder et al. 2026) all reported no significant difference in milk leptin concentrations between GDM and non‐GDM groups. However, leptin showed repeated associations with maternal adiposity. Galante et al. (2020) found higher milk leptin concentrations in overweight and obese mothers (*p* < 0.001) (Galante et al. 2020). Positive associations between HM leptin and pre‐pregnancy or postpartum BMI were also reported by other studies (Nunes et al. 2017; Yu et al. 2018; Balcells‐Esponera et al. 2024; Jin et al. 2026; Bruder et al. 2026).

Additional modifiers of HM leptin were identified in some studies. In Galante et al. (2021), maternal ethnicity was associated with HM leptin differences (*p* = 0.024), leptin concentrations were higher in singleton than in multiple pregnancies (*p* = 0.020), and cesarean delivery was associated with lower HM leptin (*p* = 0.007). The same study also demonstrated significant correlations between leptin and IGF‐1 concentrations at days 5 and 10 postpartum (*p* < 0.05) (Galante et al. 2021). In another study, leptin concentrations decreased from colostrum to mature milk in the small‐for‐gestational‐age (SGA) group (*p* = 0.050) (Nunes et al. 2017).

#### 3.2.3 Resistin

Evidence for HM resistin was very limited. Only Lis‐Kuberka et al. (2024) measured resistin directly in colostrum collected during the first 1–7 days of lactation and found no significant difference between mothers with GDM and normoglycemic controls (Lis‐Kuberka et al. 2024). Thus, although resistin was retained as a predefined target hormone because of its metabolic relevance (Ayman et al. 2019), the available HM literature remains insufficient to draw conclusions regarding its alteration in GDM.

#### 3.2.4 Other HM Hormones Reported Alongside the Three Target Adipokines

Several studies measured additional HM hormones together with adiponectin and leptin, providing broader metabolic context. Lis‐Kuberka et al. (2024) reported lower colostrum ghrelin in the diet‐treated GDM subgroup compared with the insulin‐treated GDM subgroup and controls (*p* = 0.01), while adiponectin, leptin, and resistin remained unchanged (Lis‐Kuberka et al. 2024). Choi et al. (2022) found lower milk insulin at 1 and 3 months postpartum (*p* < 0.03 and *p* < 0.003, respectively) and lower milk glucose at both time points (*p* < 0.02 and *p* < 0.01) in the GDM group, alongside higher C‐reactive protein (CRP) levels (*p* < 0.001) (Choi et al. 2022). In Yu et al. (2018), GDM mothers had higher milk insulin in both colostrum and mature milk (*p* = 0.047 and *p* = 0.021, respectively), but lower ghrelin in colostrum and mature milk at day 90 (*p* = 0.011 and *p* < 0.001, respectively) (Yu et al. 2018). Nunes et al. (2017) found no overall association between GDM and the hormonal composition of colostrum or mature milk, although insulin levels decreased from colostrum to mature milk in the SGA group (*p* = 0.012) (Nunes et al. 2017).

### 3.3 HM MiRNA Expression Profiles in GDM Mothers

**FIGURE 2:**
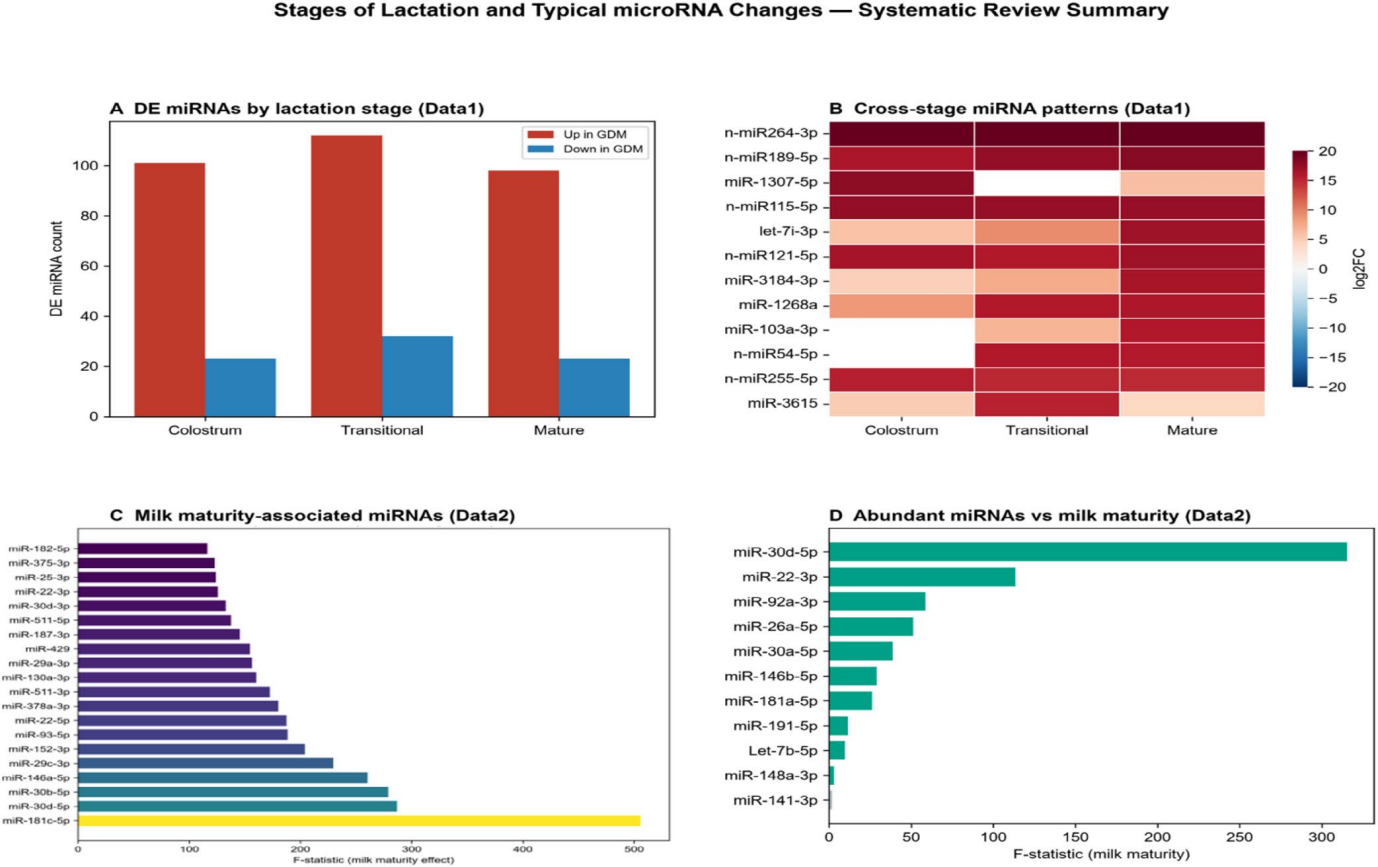
Four‐panel composite summary for the systematic review. (A) Differentially expressed miRNA counts by stage; (B) Heatmap of log_2_ fold‐change (GDM vs. Healthy) for the top recurring miRNAs across colostrum, transitional, and mature milk. Red indicates GDM upregulation; Blue indicates GDM downregulation; (C) Top 20 miRNAs ranked by F‐statistic for association with milk maturity (0, 1, 4 months post‐delivery) in the 500‐sample cohort; (D) Effect of milk maturity on the 11 most abundant breast milk miRNAs from linear mixed models. Green bars denote statistically significant effects (*p* < 0.05).

Table 2 shows a comparison of milk miRNA levels among breastfeeding mothers with GDM compared to healthy counterparts. It is important to emphasize that all associations between miRNA expression and maternal obesity or GDM reported in the included studies were correlational rather than causal. The observational nature of these studies precluded determination of whether altered miRNA expression directly contributed to GDM pathophysiology or represented a consequence of the maternal metabolic environment. Evidence on HM miRNAs in GDM was derived from three studies (Zheng et al. 2023; Hicks et al. 2022; Shah et al. 2022a). Analysis of differentially expressed exosomal miRNAs in GDM versus healthy controls across different lactation stages showed that GDM‐upregulated miRNAs predominated at all stages, with transitional milk exhibiting the highest number of dysregulated miRNAs (Figure 2A). Cross‐stage analysis revealed that *miR‐264‐3p, miR‐189‐5p*, and *miR‐115‐5p* were consistently upregulated in GDM with a log_2_ fold‐change greater than 16 across colostrum, transitional, and mature milk (Figure 2B). Consistently downregulated exosomal miRNAs in GDM included members of the *miR‐302* family (*miR‐302a‐3p, miR‐302b‐3p, and miR‐302d‐3p*), *let‐7e‐5p*, and *miR‐122‐5p* (Figure S2) (Zheng et al. 2023).

In evaluations of milk maturity‐associated miRNAs, 238 miRNAs were significantly associated with lactation stage, with the largest effects observed for *miR‐181c‐5p* (F = 506.3), *miR‐30d‐5p* (F = 287.4), *miR‐30b‐5p* (F = 279.5), and *miR‐146a‐5p* (F = 260.8) (Figure 2C). Among the 11 most abundant milk miRNAs, milk maturity had the largest effect on *miR‐30d‐5p* (F = 316, *p* < 0.05), *miR‐22‐3p* (F = 114), and *miR‐92a‐3p* (F = 59) (Figure 2D). Temporal analysis showed that GDM‐associated miRNA alterations exhibited distinct trajectories across postpartum months 0, 1, and 4, with some alterations emerging only at later time points (Figure S3) (Hicks et al. 2022).

### 3.4 MiRNA Associations With Infant Growth Outcomes

The associations between HM miRNA abundance at 1 month postpartum and infant growth outcomes at 1, 3, and 6 months were evaluated for four target miRNAs (*miR‐148a, miR‐30b, let‐7a*, and *let‐7d*) that were downregulated in GDM milk (Table 3, Figures S4–S6). A total of 16 significant associations were identified (*p* < 0.05) (Shah et al. 2022a).

**TABLE 3:** Infant outcome associations with HM miRNAs.

miRNA	Outcome age	Outcome	β (95% CI)	*p*
miR‐148a	1 month	WAZ	−0.588 (−1.125 to 0.049)	< 0.05
miR‐148a	1 month	Body fat	−3.600 (−7.115 to 0.083)	< 0.05
miR‐30b	1 month	Weight	0.253 (0.086 to 0.419)	< 0.05
miR‐30b	1 month	WAZ	0.382 (0.017 to 0.746)	< 0.05
let‐7a	3 months	Body fat	−3.038 (−5.235 to −0.840)	< 0.01
let‐7d	3 months	Body fat	2.426 (0.137 to 4.715)	< 0.05
miR‐148a	6 months	Weight	−0.489 (−0.841 to −0.136)	< 0.01
miR‐148a	6 months	Length	−1.669 (−2.557 to −0.780)	< 0.01
let‐7d	6 months	WLZ	0.652 (0.082 to 1.251)	< 0.05

At 1 month, higher breast milk *miR‐148a* abundance was significantly associated with lower infant weight‐for‐age Z‐score (WAZ; β = −0.588, 95% CI: −1.125 to −0.049; *p* < 0.05) and reduced body fat percentage (β = −3.600, 95% CI: −7.115 to −0.083; *p* < 0.05). In contrast, *miR‐30b* abundance showed positive associations with infant weight (β = 0.253, 95% CI: 0.086–0.419; *p* < 0.05) and WAZ (β = 0.382, 95% CI: 0.017–0.746; *p* < 0.05), suggesting a potential role in promoting early growth (Shah et al. 2022a).

At 3 months, body composition remained significantly associated with HM miRNA levels. Increased *let‐7a* abundance was associated with lower infant body fat percentage (β = −3.038, 95% CI: −5.235 to −0.840; *p* < 0.01), whereas *let‐7d* demonstrated the opposite pattern, showing a positive association with body fat percentage (β = 2.426, 95% CI: 0.137–4.715; *p* < 0.05) (Shah et al. 2022a).

At 6 months, *miR‐148a* remained significantly associated with infant growth, exhibiting inverse relationships with both body weight (β = −0.489, 95% CI: −0.841 to −0.136; *p* < 0.01) and body length (β = −1.669, 95% CI: −2.557 to −0.780; *p* < 0.01). Additionally, higher *let‐7d* abundance was positively associated with weight‐for‐length z‐score (WLZ; β = 0.652, 95% CI: 0.082–1.251; *p* < 0.05) (Shah et al. 2022a).

### 3.5 In Silico Regulatory Landscape and Network Topology

**FIGURE 3:**
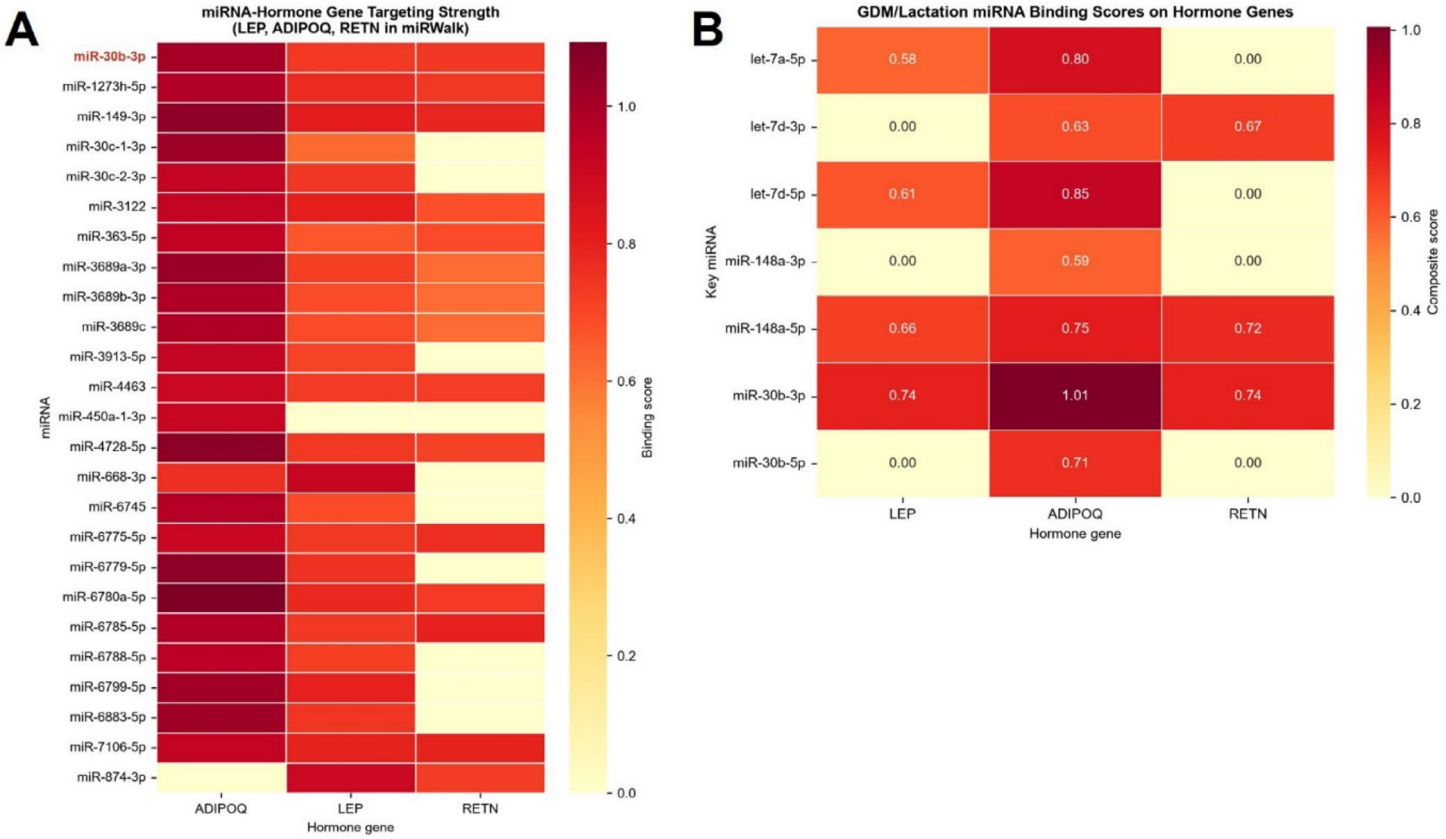
Bioinformatics analysis. (A) Heatmap of composite binding scores for the top 25 miRNAs targeting *LEP, ADIPOQ*, and *RETN*. (B) Key miRNA composite scores on hormone genes.

Computational analysis of miRNA interactions with the metabolic hormone panel identified regulatory linkages (Table S1, Figure 3A). Downstream binding analyses were restricted to target interactions for *LEP* (Leptin), *ADIPOQ* (Adiponectin), and *RETN* (Resistin) based on database availability. The eight core GDM‐ and lactation‐associated miRNAs (*miR‐148a‐5p, miR‐148a‐3p, miR‐30b‐5p, miR‐30b‐3p, let‐7a‐5p, let‐7a‐3p, let‐7d‐5p*, and *let‐7d‐3p*) exhibited high‐affinity binding scores for these genes (Table 2S, Figure 3B). Among these, *miR‐148a‐5p* and *miR‐30b‐3p* targeted all three transcripts (*LEP, ADIPOQ*, and *RETN*) (Table S2, Figure 3B). Furthermore, *miR‐30b‐3p* was confirmed to possess experimentally validated binding sites on the *ADIPOQ* transcript (Figure 3B).

**FIGURE 4:**
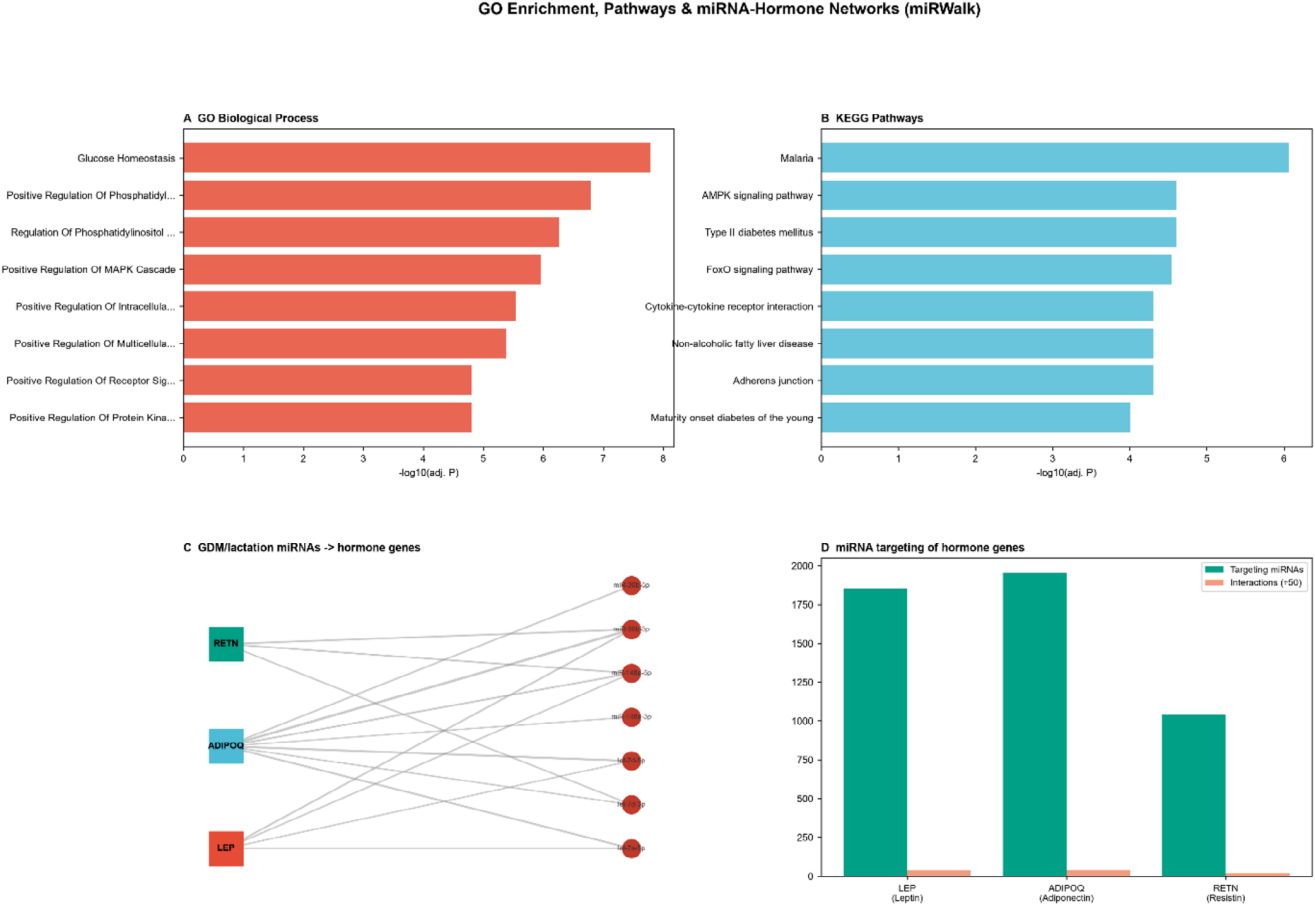
Bioinformatics analysis (A) Gene Ontology (GO) and (B) Kyoto Encyclopedia of Genes and Genomes (KEGG) pathway enrichment analysis. (C) GDM/lactation miRNA connections to *LEP, ADIPOQ, RETN*; (D) miRNA targeting counts per hormone gene.

Functional enrichment analysis of the full 27‐mRNA panel revealed significant enrichment in Glucose Homeostasis (GO:0042593, Adj. *p* = 1.61 × 10^−8^) and Regulation of Hormone Levels. Under KEGG analysis, pathways related to Malaria (Adj. *p* = 8.74 × 10^−7^) and Endocrine and other factor‐regulated calcium reabsorption were overrepresented (Figure 4A). Enrichment analysis of target genes of the eight core GDM‐associated miRNAs demonstrated significant associations with Glucose Homeostasis (Adj. *p* = 1.14 × 10^−8^), Type II Diabetes Mellitus (Adj. *p* = 2.32 × 10^−5^), and Insulin Resistance (Table S3, Figure 4B).

Bipartite network topology mapping using the top 5000 scoring miRNA‐mRNA edges identified highly connected nodes (Figure 4C). The miRNAs exhibiting the highest degree centrality were *miR‐6870‐5p, miR‐3194‐3p, miR‐6726‐5p*, and *miR‐4656* (Table S4). Within this network, the genes with the highest degree centrality were *TCF7L2, INSR, GCK*, and *HNF1A* (Table 5S). A subnetwork restricted to experimentally validated interactions confirmed that the key *let‐7* and *miR‐30* family members maintained direct regulatory edges with the adipokine genes *LEP* and *ADIPOQ* (Figure 3B, Table 4).

**TABLE 4:** GDM‐relevant miRNAs with predicted hormone gene targets.

Gene	Hormone	Targeting miRNAs (total)	Key GDM/Lactation miRNAs
*ADIPOQ*	Adiponectin	1952	*miR‐148a‐5p/3p, miR‐30b‐5p/3p, let‐7a‐5p, let‐7d‐5p/3p*
*LEP*	Leptin	1856	*miR‐148a‐5p, miR‐30b‐3p, let‐7a‐5p, let‐7d‐5p*
*RETN*	Resistin	1041	*miR‐148a‐5p, miR‐30b‐3p, let‐7d‐3p*

## 4 Discussion

This systematic review examined how gestational diabetes mellitus shapes the hormonal and miRNA composition of HM and their potential relationship to infant growth and metabolic programming. The main findings indicate that GDM‐associated changes in HM are selective rather than uniform (Lis‐Kuberka et al. 2024; Galante et al. 2020, 2021; Choi et al. 2022; Nunes et al. 2017; Yu et al. 2018; Balcells‐Esponera et al. 2024; Jin et al. 2026; Bruder et al. 2026). Among the three predefined adipokines, adiponectin showed the most consistent signal, with several studies reporting lower concentrations in GDM milk, although null findings were also common (Lis‐Kuberka et al. 2024; Galante et al. 2020, 2021; Choi et al. 2022; Nunes et al. 2017; Yu et al. 2018; Balcells‐Esponera et al. 2024; Jin et al. 2026; Bruder et al. 2026). Leptin was not consistently altered by GDM itself and appeared more strongly related to maternal adiposity (Lis‐Kuberka et al. 2024; Galante et al. 2020; Choi et al. 2022; Nunes et al. 2017; Yu et al. 2018; Balcells‐Esponera et al. 2024; Jin et al. 2026; Bruder et al. 2026). Evidence for resistin was notably sparse, with only one study finding no difference between groups (Lis‐Kuberka et al. 2024). The miRNA evidence, though limited to three studies (Zheng et al. 2023; Hicks et al. 2022; Shah et al. 2022a), suggested GDM is associated with altered exosomal miRNA profiles across lactation, with several downregulated miRNAs linked to infant anthropometric outcomes during the first 6 months (Zheng et al. 2023; Hicks et al. 2022). Bioinformatic analysis supported biological plausibility by identifying regulatory links between GDM‐associated miRNAs and adipokine genes (*LEP, ADIPOQ, RETN*), with enrichment of pathways related to glucose homeostasis, insulin resistance, and Type II diabetes mellitus. Overall, these findings support the concept that GDM may influence the milk signaling environment through coordinated changes in adipokines and miRNAs relevant to postnatal metabolic programming.

Adiponectin was the most informative of the predefined adipokines and appears to be the component most likely to reflect the metabolic disturbances associated with GDM. Studies by Galante et al. and Yu et al. reported lower adiponectin concentrations in the milk of mothers with GDM, whereas several other studies did not observe significant differences (Galante et al. 2021; Yu et al. 2018; Quinn 2021; Woo et al. 2012; Roy and Palaniyandi 2021; Wang et al. 2022). This inconsistency suggests that the association is biologically plausible yet context‐dependent rather than universal. Adiponectin is secreted predominantly by adipocytes but also by mammary glands, occurring in trimeric, hexameric, or high‐molecular‐weight (HMW) forms, with HMW being the most abundant in HM (Quinn 2021; Woo et al. 2012). Functionally, adiponectin enhances insulin sensitivity, lipid metabolism, and anti‐inflammatory effects through activation of AMP‐activated protein kinase (AMPK) and peroxisome proliferator‐activated receptor alpha (PPARα) (Woo et al. 2012; Roy and Palaniyandi 2021; Wang et al. 2022). AMPK activation, in turn, promotes fatty acid oxidation and glucose uptake while suppressing gluconeogenesis, consistent with the observation that higher adiponectin levels are generally associated with lower adiposity and improved metabolic health (Roy and Palaniyandi 2021; Wang et al. 2022; Engin 2024; Hoffstedt et al. 2004; Gavrila et al. 2003).

Within the included literature, the most compelling evidence for reduced milk adiponectin in GDM came from the longitudinal studies by Galante et al. and Yu et al. (Galante et al. 2021; Yu et al. 2018), suggesting that adiponectin may serve as a more sensitive marker of altered maternal metabolic status than the other target hormones. This finding aligns with the broader premise that adipokines reflect maternal endocrine and metabolic states and may transmit metabolic cues to the infant through milk exposure (Galante et al. 2020; Yu et al. 2018; Balcells‐Esponera et al. 2024). However, the heterogeneity across studies indicates that GDM alone is unlikely to be the sole determinant of milk adiponectin concentration. Differences in lactation stage, maternal BMI, timing of sample collection, study design, and population characteristics likely contributed to the divergent findings (Lis‐Kuberka et al. 2024; Galante et al. 2020, 2021; Choi et al. 2022; Nunes et al. 2017; Yu et al. 2018; Balcells‐Esponera et al. 2024; Jin et al. 2026; Bruder et al. 2026). Notably, adiponectin concentrations varied not only by GDM status but also by infant sex, birth weight, maternal ethnicity, and infant growth trajectories (Galante et al. 2020, 2021; Yu et al. 2018; Balcells‐Esponera et al. 2024).

The relationship between maternal adiposity and adiponectin is further complicated by bidirectional dynamics. Normal‐weight mothers exhibit a progressive decrease in adiponectin during lactation, whereas obese mothers show increased levels (Zamanillo et al. 2019). Similarly, infant adiponectin levels rise from birth to 3 months before declining during the first year (Woo et al. 2012). Some studies suggest that HM adiponectin inversely correlates with infant weight‐for‐length Z‐scores in the first 6 months (Woo et al. 2009), with higher levels potentially increasing the likelihood of exceeding growth norms at 2 years (Weyermann et al. 2007). Thus, elevated HM adiponectin in overweight mothers may exert a dual effect: protecting against early rapid catch‐up growth while potentially facilitating accelerated growth later in infancy (Woo et al. 2012; Young et al. 2018; Deierlein et al. 2011). These observations position adiponectin at the intersection of maternal metabolic dysregulation and infant developmental context, rather than functioning as a simple binary marker of GDM exposure.

In contrast to adiponectin, the overall pattern for leptin suggests that maternal body composition is a more prominent determinant than GDM status itself. Across the included studies, milk leptin was repeatedly associated with pre‐pregnancy BMI, postpartum BMI, overweight, and obesity, whereas direct differences between GDM and non‐GDM groups were generally absent (Lis‐Kuberka et al. 2024; Galante et al. 2020; Choi et al. 2022; Nunes et al. 2017; Yu et al. 2018; Balcells‐Esponera et al. 2024; Jin et al. 2026; Bruder et al. 2026). This distinction is clinically informative because GDM and maternal adiposity often coexist, yet they may influence milk composition through overlapping but non‐identical pathways. The consistent association of leptin with maternal BMI supports the view that leptin in HM reflects the maternal adipose endocrine milieu more directly than glycemic status alone (Lis‐Kuberka et al. 2024; Galante et al. 2020; Choi et al. 2022; Nunes et al. 2017; Yu et al. 2018; Balcells‐Esponera et al. 2024; Jin et al. 2026; Bruder et al. 2026). Leptin, a 16 kDa protein, is a key molecule for maternal‐fetal metabolic programming (Quinn 2021). It is secreted into HM either from maternal adipose tissue through passive diffusion or receptor‐mediated transport, or directly by lactocytes (Hassiotou et al. 2014; Suwaydi et al. 2022). Studies contrasting normal‐weight and overweight women have demonstrated a positive association between leptin and maternal BMI, insulin levels, and insulin sensitivity (Young et al. 2017). Accordingly, HM leptin in lactating obese women is roughly 2.8 times higher than in normal‐weight mothers throughout lactation (Shah et al. 2022a), a finding corroborated by other studies reporting elevated leptin concentrations in HM from obese mothers (Galante et al. 2020). Interestingly, after adjusting for GDM status and BMI, healthy mothers may exhibit higher leptin concentrations in both colostrum and mature milk compared to their GDM counterparts (Yu et al. 2018). This suggests that GDM may exert a suppressive effect on milk leptin beyond that explained by maternal adiposity alone.

Nevertheless, additional maternal factors warrant consideration. Hypertension and smoking, for instance, have been associated with lower leptin concentrations in mature milk and increased postnatal weight gain in SGA neonates (Nunes et al. 2017). Higher HM leptin, conversely, has been linked to lower infant WFL z‐scores at 4 months and 1 year, suggesting a protective role against early weight gain and childhood obesity (Nunes et al. 2017; Chan et al. 2018). This may imply that elevated HM leptin inhibits postnatal growth in SGA infants compared to their appropriate‐for‐gestational‐age peers (Dundar et al. 2005). Other modifiers, including ethnicity, singleton versus multiple pregnancy, and mode of delivery—further underscore that leptin is embedded within a broader physiological and obstetric context (Lis‐Kuberka et al. 2024; Galante et al. 2020, 2021; Choi et al. 2022; Nunes et al. 2017; Yu et al. 2018; Balcells‐Esponera et al. 2024; Jin et al. 2026; Bruder et al. 2026). Additionally, the correlation between milk leptin and IGF‐1 in early lactation suggests that leptin operates within a network of growth‐related milk signals rather than functioning as an isolated marker (Galante et al. 2021). From an interpretive standpoint, these findings imply that studies examining the effect of GDM on milk leptin without careful attention to maternal adiposity and related covariates may underestimate or misattribute sources of variation.

The miRNA component of this study extends the discussion from descriptive hormone profiling to a more integrated model of postnatal molecular signaling. The predominance of upregulated miRNAs across lactation stages, the particularly marked dysregulation in transitional milk, and the persistence of some altered miRNAs across colostrum, transitional, and mature milk collectively suggest that GDM may reshape the postnatal epigenetic cargo of milk in both stage‐dependent and sustained ways (Zheng et al. 2023; Hicks et al. 2022; Shah et al. 2022a). This observation is biologically significant because milk miRNAs are protected within extracellular vesicles (Hamdi et al. 2026; Ghafourian et al. 2026; Mo et al. 2026; Chen et al. 2025), rendering them plausible candidates for inter‐individual signaling during infancy. Although the limited number of available studies warrants caution, the consistency of direction within certain miRNA families supports the likelihood that the observed changes are biologically meaningful rather than attributable to random variation (Zheng et al. 2023; Hicks et al. 2022; Shah et al. 2022a).

The associations between selected milk miRNAs and infant growth outcomes provide some of the most compelling evidence for potential functional relevance. Downregulated miRNAs in GDM milk, including *miR‐148a, miR‐30b, let‐7a*, and *let‐7d*, were linked to infant weight, body fat percentage, body length, weight‐for‐age z‐score, and weight‐for‐length z‐score during the first 6 months (Shah et al. 2022a). In milk from normal‐weight, but not obese, mothers, several miRNAs, including *miR‐148a, let‐7a, let‐7b, miR‐103*, and *miR‐17*, were negatively correlated with adiponectin (Zamanillo et al. 2019). More specifically, lower levels of *miR‐148a* in breast milk from GDM mothers have been associated with increased infant weight and body fat percentage at 1 month. Following maternal hyperglycemia, reduced miR‐148a may affect hypothalamic regulation of satiety, thereby promoting weight gain in the newborn (Shah et al. 2022b). Conversely, *miR‐30b* overexpression has been linked to increased adipogenesis, characterized by larger lipid droplets and elevated lipogenic gene expression (Shah et al. 2022b; Hu et al. 2015). The opposing effects of miR‐148a and miR‐30b on infant adiposity suggest that their relative abundance may determine the extent of adipose tissue deposition, potentially programming long‐term obesity risk.

The *let‐7* family, which is strongly conserved across species and involved in cell growth, immune response, and neonatal development, warrants particular attention (Lee et al. 2016). More specifically, *let‐7 g‐5p* is of considerable interest as it has been linked to mammary gland metabolism and lactation and is present at elevated levels in mothers with low milk supply, a condition prevalent in GDM (Tian et al. 2019; Hicks et al. 2023). *Let‐7 g‐5p* suppresses mammary gland development and regulates milk amount and composition by targeting *HMGA2, IGF2BP1*, and *STARD13* genes, which are involved in tissue remodeling, oxidative stress regulation, and mammary epithelial cell differentiation (Hicks et al. 2023). Dysregulation of let‐7 g‐5p may therefore contribute to lactation difficulties and reduced milk production in both GDM and obese mothers. These findings suggest that altered milk miRNA abundance may relate to measurable differences in infant growth and body composition during a period of rapid developmental plasticity, reinforcing the concept that milk miRNAs function as a regulatory ensemble rather than a single‐direction growth signal.

The bioinformatic analysis strengthens this interpretation by demonstrating that several GDM‐ and lactation‐associated miRNAs have predicted or validated binding relationships with *LEP, ADIPOQ*, and *RETN*. Particularly notable was the identification of *miR‐148a‐5p* and *miR‐30b‐3p* as targeting all three adipokine‐related genes, alongside the experimentally supported interaction between *miR‐30b‐3p* and *ADIPOQ*. These findings provide a mechanistic bridge between the hormonal and miRNA branches of the review, supporting the hypothesis that part of the variability in HM adipokines may be linked to upstream posttranscriptional regulation by milk‐associated miRNAs. While such in silico evidence does not establish direct causal regulation within the lactating mammary gland or the infant, it does identify plausible molecular connections that are coherent with the observed clinical literature. The enrichment of glucose homeostasis, insulin resistance, Type II diabetes mellitus, and hormone regulation pathways further reinforces the relevance of the identified miRNA networks to the metabolic disturbances central to GDM.

The network topology analysis further situates the adipokine findings within a broader endocrine and glycemic regulatory framework already implicated in maternal metabolic dysfunction, highlighting genes such as *TCF7L2, INSR, GCK*, and *HNF1A* as highly connected nodes. These genes are central to pathways that link breastfeeding practices to metabolic outcomes. For instance, polymorphisms of *TCF7L2*, the key downstream effector of Wingless (Wnt) signaling, increase the risk of diabetes mellitus, and Wnt signaling is crucial for β‐cell development and proliferation. However, limited information exists regarding the Wnt/β‐catenin/TCF7L2‐dependent effects of breastfeeding (BF) versus formula feeding (FF) on postnatal β‐cell progenitor development, proliferation, and mass expansion. Melnik et al. (2025) support the conclusion that FF detrimentally impacts the Wnt/β‐catenin/TCF7L2‐regulated enteroendocrine‐islet axis, disrupting proper β‐cell maturation and proliferation. They propose that HM, compared to formula, offers optimized conditions for physiological Wnt signaling that promote adequate neonatal β‐cell mass expansion, potentially explaining the early diabetes‐preventive effects of prolonged BF (Melnik et al. 2025).

Additional genetic and expression studies further support these mechanistic connections. Sanchez et al. demonstrated that expression levels of *LEP‐R*, *INSR*, and *CPT1A* genes are higher in overweight children compared to normal‐weight peers (Sánchez et al. 2012). Conversely, Priego et al. showed that higher expression of *FASN, PPAR‐α*, and *INSR* genes in breastfed infants is associated with a lower risk of overweight compared to formula‐fed infants (Priego et al. 2014). Ramasammy et al. (2021) linked *GCK* gene polymorphisms to GDM, with the AA genotype and A allele conferring higher risk (Ramasammy et al. 2021). Bernardo et al. (2025) identified *HNF1A*, which encodes transcription factor hepatocyte nuclear factor‐1 alpha, as the most commonly mutated gene in Mendelian diabetes, with loss‐ or gain‐of‐function coding variants predisposing to or protecting against polygenic T2D, respectively. They demonstrated that diabetes arises from β cell‐autonomous defects and identified direct β cell genomic targets of *HNF1A*, uncovering a regulatory axis where *HNF1A* controls transcription of *A1CF*, which orchestrates an RNA splicing program encompassing genes that regulate β cell function. This *HNF1A‐A1CF* transcription‐splicing axis is suppressed in β cells from T2D individuals, while genetic variants reducing pancreatic islet A1CF are associated with increased glycemia and T2D susceptibility. These findings identify a linear hierarchy that coordinates β cell‐specific transcription and splicing programs and link this pathway to T2D pathogenesis (Bernardo et al. 2025).

Although these findings support our bioinformatics prediction, we should emphasize that the identified miRNA‐target‐hormone relationships were primarily hypothesis‐generating; therefore, they should not be interpreted as causal without direct experimental validation.

## 5 Limitations

Several limitations should be considered when interpreting the findings of this systematic review. First, the evidence base was relatively small, particularly for miRNA analyses (*n* = 3) and resistin (*n* = 1), which limits the breadth and generalizability of the conclusions. The considerable heterogeneity across included studies, in terms of sample type (colostrum versus mature milk), lactation stage, timing of collection, GDM diagnostic criteria, participant characteristics, and analytical methods, precluded quantitative meta‐analysis and likely contributed to inconsistent findings. Second, several included studies relied on hospital records or self‐reported clinical information rather than standardized prospective ascertainment, potentially introducing misclassification bias. Third, the observational nature of the underlying evidence limits causal inference, especially regarding associations between milk components and infant growth outcomes. Fourth, important effect modifiers, including maternal BMI, ethnicity, mode of delivery, smoking status, and pregnancy characteristics, varied substantially between studies and were not uniformly adjusted for, making it difficult to isolate the independent effect of GDM. Fifth, the bioinformatics network presented in this study was intended to identify potentially relevant regulatory relationships and to generate testable hypotheses rather than to establish direct molecular mechanisms. Although several predicted interactions involved biologically plausible targets, these associations did not necessarily correspond to experimentally validated pathways in the context of HM, GDM, or infant metabolic programming. Therefore, all inferred miRNA‐target‐hormone relationships should be interpreted with caution until confirmed in functional studies. Finally, the restriction to English‐language publications may have introduced language bias, and the exclusion of unpublished data may have contributed to publication bias.

## 6 Conclusions

This systematic review demonstrates that GDM is associated with selective alterations in the hormonal and miRNA composition of HM. Adiponectin emerged as the most consistently affected adipokine, though its association with GDM appears context‐dependent rather than universal. Leptin reflects maternal adiposity more reliably than GDM status itself, while resistin remains insufficiently characterized. The miRNA evidence, though limited, suggests GDM may reshape the epigenetic cargo of milk in stage‐dependent ways, with several downregulated miRNAs linked to infant growth outcomes during the first 6 months of life. Bioinformatics further supports biological plausibility by identifying regulatory connections between GDM‐associated miRNAs and adipokine genes, with enrichment of pathways related to glucose homeostasis and insulin resistance.

Clinically, these findings suggest that the metabolic effects of breastfeeding in GDM‐exposed dyads may depend on milk's molecular composition beyond duration or exclusivity. However, current evidence does not support routine clinical use of these molecules as biomarkers. Future research should prioritize larger longitudinal studies with standardized protocols, simultaneous measurement of adipokines and miRNAs, and experimental validation of the regulatory links identified here. Such efforts will determine whether these molecular alterations are merely correlates of maternal metabolic status or active contributors to early‐life metabolic programming, ultimately informing strategies to optimize infant nutrition in GDM‐affected pregnancies.

## Author Contributions

**Maryam Davoudi:** writing – original draft, investigation, methodology. **Zhijun Zhang:** writing – original draft, visualization, conceptualization. **Mojde Ahmadi:** writing – original draft, methodology. **Reza Afrisham:** writing – review and editing, supervision, conceptualization. **Parmida Dehghan:** writing – original draft, methodology. **Amirreza Ghafourian:** writing – original draft, methodology, data curation. **Hamid Choobineh:** writing – review and editing, supervision, conceptualization. **Xiaolei Miao:** writing – review and editing, supervision, conceptualization. **Seyed Mohammad Ayyoubzadeh:** methodology.

## Funding

This project was supported by the Doctoral Research Fund of Hubei University of Science and Technology (project No. Q201810) and Hubei University of Science and Technology Horizontal Project (2024HX140).

## Ethics Statement

The authors have nothing to report.

## Consent

All authors agree to publish this review.

## Conflicts of Interest

The authors declare no conflicts of interest.

## Supporting information


**Data S1:** fsn372256‐sup‐0001‐Supplmentaryfiles.zip.

**Figure S1:** Bias assessment results.
**Figure S2:** Diverging bar charts of the top upregulated (red) and downregulated (blue) exosomal miRNAs per lactation stage. Novel miRNAs dominate the upregulated set; miR‐302 family and let‐7 members dominate downregulation.
**Figure S3:** Line plots of log2 fold‐change (GDM vs. control) for the 12 most temporally variable miRNAs across 0, 1, and 4 months postpartum (Data 2, Table S12).
**Figure S4:** Composite figure for Table IV. (A) GDM context showing four miRNAs reduced in GDM milk; (B–D) forest plots of β coefficients and 95% CIs for all miRNA–outcome associations at 1, 3, and 6 months. Square markers = *p* < 0.05; diamond = *p* < 0.01.
**Figure S5:** Forest plot of the 16 statistically significant associations between 1‐month milk exosomal miRNA abundance and subsequent infant outcomes. miR‐148a shows predominantly negative associations with growth and adiposity; miR‐30b shows positive associations at 1 month.
**Figure S6:** Heatmap of standardized β coefficients for all miRNA–outcome combinations across 1, 3, and 6‐month assessment ages. Asterisks denote *p* < 0.05 (*), *p* < 0.01 (**).
**Table S1:** Binding Evidence by Hormone Gene.
**Table S2:** GDM/Lactation miRNA Targeting.
**Table S3:** Pathway Enrichment.
**Table S4:** Top hub miRNAs.
**Table S5:** Top hub genes.

## Data Availability

Kindly see the Supporting Information for data on miRNA datasets.

## References

[fsn372256-bib-0001] Alsaweed, M. , C. T. Lai , P. E. Hartmann , D. T. Geddes , and F. Kakulas . 2016. “Human Milk miRNAs Primarily Originate From the Mammary Gland Resulting in Unique miRNA Profiles of Fractionated Milk.” Scientific Reports 6, no. 1: 20680. 26854194 10.1038/srep20680 PMC4745068

[fsn372256-bib-0002] Amirian, A. , F. A. Rahnemaei , and F. Abdi . 2020. “Role of C‐Reactive Protein (CRP) or High‐Sensitivity CRP in Predicting Gestational Diabetes Mellitus: Systematic Review.” Diabetes and Metabolic Syndrome: Clinical Research and Reviews 14, no. 3: 229–236. 10.1016/j.dsx.2020.02.004 32247209

[fsn372256-bib-0003] Ayman, S. S. , T. Mohamed , M. J. Amira , I. R. Wafaa , M. D. Abd‐Elreheem , and T. Noha . 2019. “The Association Between Resistin, Leptin and Adiponectin With Obesity and Type 2 Diabetes Mellitus.” Medical Journal of Cairo University 87, no. December: 4227–4237.

[fsn372256-bib-0004] Balcells‐Esponera, C. , C. Borràs‐Novell , M. López‐Abad , et al. 2024. “Bioactive Peptides in Preterm Human Milk: Impact of Maternal Characteristics and Their Association to Neonatal Outcomes.” BioFactors 50, no. 1: 135–144. 37584623 10.1002/biof.1997

[fsn372256-bib-0005] Barbour, L. A. 2019. “Metabolic Culprits in Obese Pregnancies and Gestational Diabetes Mellitus: Big Babies, Big Twists, Big Picture: The 2018 Norbert Freinkel Award Lecture.” Diabetes Care 42, no. 5: 718–726. 31010942 10.2337/dci18-0048 PMC6489109

[fsn372256-bib-0006] Bernardo, E. , M. G. De Vas , D. Balboa , et al. 2025. “HNF1A and A1CF Coordinate a beta Cell Transcription‐Splicing axis That Is Disrupted in Type 2 Diabetes.” Cell Metabolism 37, no. 9: 1870–1889. 40774250 10.1016/j.cmet.2025.07.007 PMC12714141

[fsn372256-bib-0007] Bruder, A. , L. Ellsworth , J. Sturza , B. Gregg , A. L. Miller , and J. C. Lumeng . 2026. “The Association of Human Milk Appetite‐Regulating Hormones With Infant Growth and Eating Behaviors to Age Six Months.” Nutrients 18, no. 8: 1203. 42075016 10.3390/nu18081203 PMC13118950

[fsn372256-bib-0008] Chan, D. , S. Goruk , A. B. Becker , et al. 2018. “Adiponectin, Leptin and Insulin in Breast Milk: Associations With Maternal Characteristics and Infant Body Composition in the First Year of Life.” International Journal of Obesity 42, no. 1: 36–43. 28925410 10.1038/ijo.2017.189

[fsn372256-bib-0009] Chen, L. , F. Amraee , S. Sadegh‐Nejadi , et al. 2025. “Molecular Mechanisms Linking Adipose Tissue‐Derived Small Extracellular Vesicles/Exosomes to the Development or Amelioration of Obesity, Insulin Resistance, and Diabetes‐Related Complications.” European Journal of Medical Research 30, no. 1: 1049. 41174795 10.1186/s40001-025-03288-7 PMC12577357

[fsn372256-bib-0010] Choi, Y. , E. M. Nagel , H. Kharoud , et al. 2022. “Gestational Diabetes Mellitus Is Associated With Differences in Human Milk Hormone and Cytokine Concentrations in a Fully Breastfeeding United States Cohort.” Nutrients 14, no. 3: 667. 35277026 10.3390/nu14030667 PMC8838140

[fsn372256-bib-0011] Deierlein, A. L. , A. M. Siega‐Riz , L. S. Adair , and A. H. Herring . 2011. “Effects of Pre‐Pregnancy Body Mass Index and Gestational Weight Gain on Infant Anthropometric Outcomes.” Journal of Pediatrics 158, no. 2: 221–226. 20863516 10.1016/j.jpeds.2010.08.008 PMC3017634

[fsn372256-bib-0012] Di, S. 2014. “Diagnosis and classification of diabetes mellitus.” Diabetes Care 37, no. 1: S81–S90. 24357215 10.2337/dc14-S081

[fsn372256-bib-0013] Dundar, N. O. , O. Anal , B. Dundar , H. Ozkan , S. Caliskan , and A. Büyükgebiz . 2005. “Longitudinal Investigation of the Relationship Between Breast Milk Leptin Levels and Growth in Breast‐Fed Infants.” Journal of Pediatric Endocrinology & Metabolism 18, no. 2: 181–188. 15751607 10.1515/jpem.2005.18.2.181

[fsn372256-bib-0014] Engin, A. 2024. “Adiponectin Resistance in Obesity: Adiponectin Leptin/Insulin Interaction.” Obesity and Lipotoxicity 2024, no. 1460: 431–462. 10.1007/978-3-031-63657-8_15 39287861

[fsn372256-bib-0015] Farahvar, S. , A. Walfisch , and E. Sheiner . 2019. “Gestational Diabetes Risk Factors and Long‐Term Consequences for Both Mother and Offspring: A Literature Review.” Expert Review of Endocrinology & Metabolism 14, no. 1: 63–74. 30063409 10.1080/17446651.2018.1476135

[fsn372256-bib-0016] Galante, L. , H. Lagström , M. H. Vickers , et al. 2020. “Sexually Dimorphic Associations Between Maternal Factors and Human Milk Hormonal Concentrations.” Nutrients 12, no. 1: 152. 31935821 10.3390/nu12010152 PMC7019968

[fsn372256-bib-0017] Galante, L. , C. M. Reynolds , A. M. Milan , et al. 2021. “Preterm Human Milk: Associations Between Perinatal Factors and Hormone Concentrations Throughout Lactation.” Pediatric Research 89, no. 6: 1461–1469. 32726796 10.1038/s41390-020-1069-1

[fsn372256-bib-0018] Gao, M. , S. Cao , N. Li , et al. 2022. “Risks of Overweight in the Offspring of Women With Gestational Diabetes at Different Developmental Stages: A meta‐Analysis With More Than Half a Million Offspring.” Obesity Reviews 23, no. 3: e13395. 34820996 10.1111/obr.13395

[fsn372256-bib-0019] Gavrila, A. , J. L. Chan , N. Yiannakouris , et al. 2003. “Serum Adiponectin Levels Are Inversely Associated With Overall and Central Fat Distribution but Are Not Directly Regulated by Acute Fasting or Leptin Administration in Humans: Cross‐Sectional and Interventional Studies.” Journal of Clinical Endocrinology & Metabolism 88, no. 10: 4823–4831. 14557461 10.1210/jc.2003-030214

[fsn372256-bib-0020] Ghafourian, A. R. , A. S. Mohseni , R. Ghasemi , et al. 2026. “Preclinical Evidence of Extracellular Vesicle‐Derived MicroRNAs in Relieving Diabetic Peripheral Neuropathy: A Systematic Review.” Journal of Molecular Neuroscience 76, no. 2: 78. 42095965 10.1007/s12031-026-02500-5

[fsn372256-bib-0021] Gunderson, E. P. , L. C. Greenspan , M. S. Faith , S. R. Hurston , C. P. Quesenberry Jr. , and Investigators SOS . 2018. “Breastfeeding and Growth During Infancy Among Offspring of Mothers With Gestational Diabetes Mellitus: A Prospective Cohort Study.” Pediatric Obesity 13, no. 8: 492–504. 29691992 10.1111/ijpo.12277 PMC6108892

[fsn372256-bib-0022] Hamdi, M. , A. Rahimizadeh , M. Ahmadi , et al. 2026. “A Systematic Review of Urinary Extracellular Vesicle‐Derived Non‐Coding RNAs in Diabetic Nephropathy: Expression Profiles, Clinical Correlations, and Diagnostic Performance.” Molecular Biology Reports 53, no. 1: 359. 41636920 10.1007/s11033-026-11523-5

[fsn372256-bib-0023] Hassiotou, F. , D. Savigni , P. Hartmann , and D. Geddes . 2014. “Mammary Cells Synthesize Appetite Hormones That May Contribute to Breastmilk (38.8).” FASEB Journal 28: 38.

[fsn372256-bib-0024] Hicks, S. D. , D. Chandran , A. Confair , A. Ward , and S. L. Kelleher . 2023. “Human Milk‐Derived Levels of Let‐7g‐5p May Serve as a Diagnostic and Prognostic Marker of Low Milk Supply in Breastfeeding Women.” Nutrients 15, no. 3: 567. 36771276 10.3390/nu15030567 PMC9920885

[fsn372256-bib-0025] Hicks, S. D. , A. Confair , K. Warren , and D. Chandran . 2022. “Levels of Breast Milk MicroRNAs and Other Non‐Coding RNAs Are Impacted by Milk Maturity and Maternal Diet.” Frontiers in Immunology 12: 785217. 35095859 10.3389/fimmu.2021.785217 PMC8796169

[fsn372256-bib-0026] Hoffstedt, J. , E. Arvidsson , E. Sjölin , K. Wåhlén , and P. Arner . 2004. “Adipose Tissue Adiponectin Production and Adiponectin Serum Concentration in Human Obesity and Insulin Resistance.” Journal of Clinical Endocrinology & Metabolism 89, no. 3: 1391–1396. 15001639 10.1210/jc.2003-031458

[fsn372256-bib-0027] Hu, F. , M. Wang , T. Xiao , et al. 2015. “miR‐30 Promotes Thermogenesis and the Development of Beige Fat by Targeting RIP140.” Diabetes 64, no. 6: 2056–2068. 25576051 10.2337/db14-1117 PMC4876748

[fsn372256-bib-0028] Isganaitis, E. , S. Venditti , T. J. Matthews , C. Lerin , E. W. Demerath , and D. A. Fields . 2019. “Maternal Obesity and the Human Milk Metabolome: Associations With Infant Body Composition and Postnatal Weight Gain.” American Journal of Clinical Nutrition 110, no. 1: 111–120. 30968129 10.1093/ajcn/nqy334 PMC6599743

[fsn372256-bib-0029] Jin, X. , A. H. Warden , C. T. Lai , et al. 2026. “Human Milk Composition Is Associated With Maternal Overweight/Obesity and Low Milk Supply, With Implications for Infant Weight Outcomes: A Cross‐Sectional Study.” American Journal of Clinical Nutrition 123, no. 1: 101098. 41235974 10.1016/j.ajcnut.2025.10.015

[fsn372256-bib-0030] Karagoz, Z. K. , S. Aydin , K. Ugur , et al. 2022. “Molecular Communication Between Apelin‐13, Apelin‐36, Elabela, and Nitric Oxide in Gestational Diabetes Mellitus.” European Review for Medical and Pharmacological Sciences 26, no. 9: 3289–3300. 35587081 10.26355/eurrev_202205_28748

[fsn372256-bib-0031] Kc, K. , S. Shakya , and H. Zhang . 2015. “Gestational Diabetes Mellitus and Macrosomia: A Literature Review.” Annals of Nutrition and Metabolism 66, no. 2: 14–20. 10.1159/000371628 26045324

[fsn372256-bib-0032] Kong, L. , X. Chen , M. Gissler , and C. Lavebratt . 2020. “Relationship of Prenatal Maternal Obesity and Diabetes to Offspring Neurodevelopmental and Psychiatric Disorders: A Narrative Review.” International Journal of Obesity 44, no. 10: 1981–2000. 32494038 10.1038/s41366-020-0609-4 PMC7508672

[fsn372256-bib-0033] Lee, H. , S. Han , C. S. Kwon , and D. Lee . 2016. “Biogenesis and Regulation of the Let‐7 miRNAs and Their Functional Implications.” Protein & Cell 7, no. 2: 100–113. 26399619 10.1007/s13238-015-0212-y PMC4742387

[fsn372256-bib-0034] Lis‐Kuberka, J. , M. Berghausen‐Mazur , and M. Orczyk‐Pawiłowicz . 2024. “Gestational Diabetes Mellitus and Colostral Appetite‐Regulating Adipokines.” International Journal of Molecular Sciences 25, no. 7: 3853. 38612666 10.3390/ijms25073853 PMC11011253

[fsn372256-bib-0035] Melnik, B. C. , R. Weiskirchen , S. Weiskirchen , et al. 2025. “Diabetes‐Preventive Molecular Mechanisms of Breast Versus Formula Feeding: New Insights Into the Impact of Milk on Stem Cell Wnt Signaling.” Frontiers in Nutrition 12: 1652297. 40799516 10.3389/fnut.2025.1652297 PMC12341479

[fsn372256-bib-0036] Mirra, P. , C. Nigro , I. Prevenzano , et al. 2018. “The Destiny of Glucose From a microRNA Perspective.” Frontiers in Endocrinology 9: 46. 29535681 10.3389/fendo.2018.00046 PMC5834423

[fsn372256-bib-0037] Mo, Q. , A. Ghafourian , M. Hamdi , et al. 2026. “Consolidating Clinical Insights and Uncovering Novel Regulatory Mechanisms of Exosomal MicroRNAs in Obesity and Metabolic Dysfunction Associated Steatotic Liver Disease: A Systematic Review and Bioinformatics Analysis.” Food Science & Nutrition 14, no. 5: e71901. 42254426 10.1002/fsn3.71901 PMC13238684

[fsn372256-bib-0038] Murray, C. J. L. , A. Y. Aravkin , P. Zheng , et al. 2020. “Global Burden of 87 Risk Factors in 204 Countries and Territories, 1990–2019: A Systematic Analysis for the Global Burden of Disease Study 2019.” Lancet 396, no. 10258: 1223–1249. 33069327 10.1016/S0140-6736(20)30752-2 PMC7566194

[fsn372256-bib-0039] Nijs, H. , and K. Benhalima . 2020. “Gestational Diabetes Mellitus and the Long‐Term Risk for Glucose Intolerance and Overweight in the Offspring: A Narrative Review.” Journal of Clinical Medicine 9, no. 2: 599. 32098435 10.3390/jcm9020599 PMC7074239

[fsn372256-bib-0040] Nunes, M. , C. H. da Silva , V. L. Bosa , J. R. Bernardi , I. C. R. Werlang , and M. Z. Goldani . 2017. “Could a Remarkable Decrease in Leptin and Insulin Levels From Colostrum to Mature Milk Contribute to Early Growth Catch‐Up of SGA Infants?” BMC Pregnancy and Childbirth 17, no. 1: 410. 29212463 10.1186/s12884-017-1593-0 PMC5719575

[fsn372256-bib-0041] Ornoy, A. , M. Becker , L. Weinstein‐Fudim , and Z. Ergaz . 2021. “Diabetes During Pregnancy: A Maternal Disease Complicating the Course of Pregnancy With Long‐Term Deleterious Effects on the Offspring. A Clinical Review.” International Journal of Molecular Sciences 22, no. 6: 2965. 33803995 10.3390/ijms22062965 PMC7999044

[fsn372256-bib-0042] Page, M. J. , J. E. McKenzie , P. M. Bossuyt , et al. 2021. “The PRISMA 2020 Statement: An Updated Guideline for Reporting Systematic Reviews.” BMJ 372: n71. 33782057 10.1136/bmj.n71 PMC8005924

[fsn372256-bib-0043] Pathirana, M. M. , Z. S. Lassi , C. T. Roberts , and P. H. Andraweera . 2020. “Cardiovascular Risk Factors in Offspring Exposed to Gestational Diabetes Mellitus in Utero: Systematic Review and meta‐Analysis.” Journal of Developmental Origins of Health and Disease 11, no. 6: 599–616. 31902382 10.1017/S2040174419000850

[fsn372256-bib-0044] Priego, T. , J. Sánchez , C. Picó , et al. 2014. “Influence of Breastfeeding on Blood‐Cell Transcript‐Based Biomarkers of Health in Children.” Pediatric Obesity 9, no. 6: 463–470. 24277691 10.1111/j.2047-6310.2013.00204.x

[fsn372256-bib-0045] Quinn, E. A. 2021. “Centering Human Milk Composition as Normal Human Biological Variation.” American Journal of Human Biology 33, no. 1: e23564. 33432701 10.1002/ajhb.23564

[fsn372256-bib-0046] Ramasammy, R. , L. Munisammy , K. Sweta , et al. 2021. “Association Between GCK Gene Polymorphism and Gestational Diabetes Mellitus and Its Pregnancy Outcomes.” Meta Gene 28: 100856.

[fsn372256-bib-0047] Roy, B. , and S. S. Palaniyandi . 2021. “Tissue‐Specific Role and Associated Downstream Signaling Pathways of Adiponectin.” Cell & Bioscience 11, no. 1: 77. 33902691 10.1186/s13578-021-00587-4 PMC8073961

[fsn372256-bib-0048] Saliminejad, K. , H. R. Khorram Khorshid , S. Soleymani Fard , and S. H. Ghaffari . 2019. “An Overview of microRNAs: Biology, Functions, Therapeutics, and Analysis Methods.” Journal of Cellular Physiology 234, no. 5: 5451–5465. 30471116 10.1002/jcp.27486

[fsn372256-bib-0049] Sánchez, J. , T. Priego , C. Picó , et al. 2012. “Blood Cells as a Source of Transcriptional Biomarkers of Childhood Obesity and Its Related Metabolic Alterations: Results of the IDEFICS Study.” Journal of Clinical Endocrinology and Metabolism 97, no. 4: E648–E652. 22278432 10.1210/jc.2011-2209

[fsn372256-bib-0050] Shah, K. B. , S. D. Chernausek , L. D. Garman , et al. 2021. “Human Milk Exosomal MicroRNA: Associations With Maternal Overweight/Obesity and Infant Body Composition at 1 Month of Life.” Nutrients 13, no. 4: 1091. 33801634 10.3390/nu13041091 PMC8066780

[fsn372256-bib-0051] Shah, K. B. , D. A. Fields , N. P. Pezant , et al. 2022a. “Gestational Diabetes Mellitus Is Associated With Altered Abundance of Exosomal MicroRNAs in Human Milk.” Clinical Therapeutics 44, no. 2: 172–185. 35090750 10.1016/j.clinthera.2022.01.005 PMC9089438

[fsn372256-bib-0052] Shah, K. B. , D. A. Fields , N. P. Pezant , et al. 2022b. “Gestational Diabetes Mellitus Is Associated With Altered Abundance of Exosomal MicroRNAs in Human Milk.” Clinical Therapeutics 44, no. 2: 172–185. 35090750 10.1016/j.clinthera.2022.01.005 PMC9089438

[fsn372256-bib-0053] Skrypnik, D. , P. Bogdański , A. Zawiejska , and E. Wender‐Ożegowska . 2019. “Role of Gestational Weight Gain, Gestational Diabetes, Breastfeeding, and Hypertension in Mother‐To‐Child Obesity Transmission.” Pol Arch Intern Med 129, no. 4: 267–275. 30688285 10.20452/pamw.4426

[fsn372256-bib-0054] Suwaydi, M. A. , X. Zhou , S. L. Perrella , et al. 2022. “The Impact of Gestational Diabetes Mellitus on Human Milk Metabolic Hormones: A Systematic Review.” Nutrients 14, no. 17: 3620. 36079876 10.3390/nu14173620 PMC9460195

[fsn372256-bib-0055] Tian, L. , Y. Li , C. Wang , and Q. Li . 2019. “Let‐7g‐5p Regulates Mouse Mammary Cells Differentiation and Function by Targeting PRKCA.” Journal of Cellular Physiology 234, no. 7: 10101–10110. 30422318 10.1002/jcp.27676

[fsn372256-bib-0056] Van Syoc, E. , M. Stegman , R. Sullivan , A. Confair , K. Warren , and S. D. Hicks . 2024. “Associations of Maternal Breastmilk microRNAs and Infant Obesity Status at 1 Year.” Genes 15, no. 6: 813. 38927748 10.3390/genes15060813 PMC11203006

[fsn372256-bib-0057] Wang, D. , L. Yang , and Y. Liu . 2022. “Targeting AMPK Signaling in the Liver: Implications for Obesity and Type 2 Diabetes Mellitus.” Current Drug Targets 23, no. 11: 1057–1071. 36028937 10.2174/1389450123666220429082702

[fsn372256-bib-0058] Weir, T. L. , M. Majumder , and S. J. Glastras . 2024. “A Systematic Review of the Effects of Maternal Obesity on Neonatal Outcomes in Women With Gestational Diabetes.” Obesity Reviews 25: e13747. 38679418 10.1111/obr.13747

[fsn372256-bib-0059] Weyermann, M. , H. Brenner , and D. Rothenbacher . 2007. “Adipokines in Human Milk and Risk of Overweight in Early Childhood: A Prospective Cohort Study.” Epidemiology 18, no. 6: 722–729. 18062063 10.1097/ede.0b013e3181567ed4

[fsn372256-bib-0060] Woo, J. G. , M. L. Guerrero , M. Altaye , et al. 2009. “Human Milk Adiponectin Is Associated With Infant Growth in Two Independent Cohorts.” Breastfeeding Medicine 4, no. 2: 101–109. 19500050 10.1089/bfm.2008.0137 PMC2779028

[fsn372256-bib-0061] Woo, J. G. , M. L. Guerrero , F. Guo , et al. 2012. “Human Milk Adiponectin Affects Infant Weight Trajectory During the Second Year of Life.” Journal of Pediatric Gastroenterology and Nutrition 54, no. 4: 532–539. 22094897 10.1097/MPG.0b013e31823fde04 PMC3299902

[fsn372256-bib-0062] Young, B. E. , C. Levek , R. M. Reynolds , et al. 2018. “Bioactive Components in Human Milk Are Differentially Associated With Rates of Lean and Fat Mass Deposition in Infants of Mothers With Normal vs. Elevated BMI.” Pediatric Obesity 13, no. 10: 598–606. 30092608 10.1111/ijpo.12394 PMC6390491

[fsn372256-bib-0063] Young, B. E. , Z. Patinkin , C. Palmer , et al. 2017. “Human Milk Insulin Is Related to Maternal Plasma Insulin and BMI: But Other Components of Human Milk Do Not Differ by BMI.” European Journal of Clinical Nutrition 71, no. 9: 1094–1100. 28513622 10.1038/ejcn.2017.75 PMC5587359

[fsn372256-bib-0064] Yu, X. , S. S. Rong , X. Sun , et al. 2018. “Associations of Breast Milk Adiponectin, Leptin, Insulin and Ghrelin With Maternal Characteristics and Early Infant Growth: A Longitudinal Study.” British Journal of Nutrition 120, no. 12: 1380–1387. 30375294 10.1017/S0007114518002933

[fsn372256-bib-0065] Zamanillo, R. , J. Sánchez , F. Serra , and A. Palou . 2019. “Breast Milk Supply of Microrna Associated With Leptin and Adiponectin Is Affected by Maternal Overweight/Obesity and Influences Infancy Bmi.” Nutrients 11, no. 11: 2589. 31661820 10.3390/nu11112589 PMC6893542

[fsn372256-bib-0066] Zehravi, M. , M. Maqbool , and I. Ara . 2021. “Correlation Between Obesity, Gestational Diabetes Mellitus, and Pregnancy Outcomes: An Overview.” International Journal of Adolescent Medicine and Health 33, no. 6: 339–345. 34142511 10.1515/ijamh-2021-0058

[fsn372256-bib-0067] Zheng, Z. , J. Mo , F. Lin , et al. 2023. “Milk Exosomes From Gestational Diabetes Mellitus (GDM) and Healthy Parturient Exhibit Differential miRNAs Profiles and Distinct Regulatory Bioactivity on Hepatocyte Proliferation.” Molecular Nutrition & Food Research 67, no. 16: e2300005. 37357556 10.1002/mnfr.202300005

