# Supplementary material for: Alterations in Human Milk Adiponectin, Leptin, Resistin, and microRNAs in Mothers With Gestational Diabetes Mellitus Compared With Healthy Controls: A Systematic Review and Network‐Based Analysis
Source: Food Sci Nutr. 2026 Aug 31;14(9):e72256. doi: 10.1002/fsn3.72256 (PMC13528237; doi:10.1002/fsn3.72256)
Supplement: Supplementary file 1 [file FSN3-14-e72256-s001.docx]

Table 1S. Binding Evidence by Hormone Gene

| **Gene** | **miRNAs** | **Sites** | **3′UTR** | **CDS** | **5′UTR** | **Seed** | **Validated** |
| --- | --- | --- | --- | --- | --- | --- | --- |
| Leptin (*LEP*) | 1,856 | 3,169 | 80.0% | 19.0% | 1.0% | 52.4% | 0.0% |
| Adiponectin (*ADIPOQ*) | 1,952 | 5,595 | 74.1% | 21.8% | 4.1% | 53.9% | 1.7% |
| Resistin (*RETN*) | 1,041 | 2,041 | 23.7% | 56.9% | 19.4% | 54.4% | 0.0% |

Table 2S. GDM/Lactation miRNA Targeting

| **NA** | **Gene** | **Hormone** | **Score** | **Sites** | **Validated** | **DB support** | **Tier** |
| --- | --- | --- | --- | --- | --- | --- | --- |
| *miR-30b-3p* | ADIPOQ | Adiponectin | 1.007 | 4 | Yes | Yes | very_strong |
| *let-7d-5p* | ADIPOQ | Adiponectin | 0.853 | 6 | No | No | strong |
| *let-7a-5p* | ADIPOQ | Adiponectin | 0.797 | 4 | No | No | strong |
| *miR-148a-5p* | ADIPOQ | Adiponectin | 0.750 | 3 | No | No | strong |
| *miR-30b-5p* | ADIPOQ | Adiponectin | 0.707 | 2 | No | No | good |
| *let-7d-3p* | ADIPOQ | Adiponectin | 0.630 | 2 | No | No | good |
| *miR-148a-3p* | ADIPOQ | Adiponectin | 0.593 | 1 | No | No | good |
| *miR-30b-3p* | LEP | Leptin | 0.736 | 2 | No | No | good |
| *miR-148a-5p* | LEP | Leptin | 0.663 | 1 | No | No | good |
| *let-7d-5p* | LEP | Leptin | 0.614 | 1 | No | No | good |
| *let-7a-5p* | LEP | Leptin | 0.583 | 1 | No | No | good |
| *miR-30b-3p* | RETN | Resistin | 0.739 | 3 | No | No | good |
| *miR-148a-5p* | RETN | Resistin | 0.715 | 1 | No | No | good |
| *let-7d-3p* | RETN | Resistin | 0.672 | 1 | No | No | good |

### Table 3S. Pathway Enrichment

| **Analysis** | **Top GO term** | **Adj. P** | **Top KEGG pathway** | **Adj. P** |
| --- | --- | --- | --- | --- |
| Full 27-gene panel | Glucose Homeostasis | 1.61e-08 | Malaria | 8.74e-07 |
| Key-miRNA target genes | Glucose Homeostasis | 1.14e-08 | Type II diabetes mellitus | 2.32e-05 |

### Hub Centrality

#### Table 4S. Top hub miRNAs

| **miRNA** | **Degree** |
| --- | --- |
| *miR-6870-5p* | 12 |
| *miR-3194-3p* | 10 |
| *miR-6726-5p* | 9 |
| *miR-4656* | 9 |
| *miR-4508* | 9 |
| *miR-378g* | 9 |
| *miR-4669* | 8 |
| *miR-6857-5p* | 8 |
| *miR-1275* | 8 |
| *miR-8077* | 8 |

#### Table 5S. Top hub genes

| **Gene** | **Degree** |
| --- | --- |
| *TCF7L2* | 973 |
| *SRRM1* | 644 |
| *DCN* | 564 |
| *MET* | 304 |
| *PLAT* | 274 |
| *INHBA* | 265 |
| *GCK* | 252 |
| *HNF1A* | 228 |
| *INSR* | 219 |
| *GH1* | 188 |

Figure 1S. Bias assessment results.

Figure 2S. Diverging bar charts of the top upregulated (red) and downregulated (blue) exosomal miRNAs per lactation stage. Novel miRNAs dominate the upregulated set; miR-302 family and let-7 members dominate downregulation.

Figure 3S. Line plots of log_2_ fold-change (GDM vs control) for the 12 most temporally variable miRNAs across 0, 1, and 4 months postpartum (Data2, Table S12).

Figure 4S. Composite figure for Table IV. (A) GDM context showing four miRNAs reduced in GDM milk; (B–D) forest plots of β coefficients and 95% CIs for all miRNA–outcome associations at 1, 3, and 6 months. Square markers = p < 0.05; diamond = p < 0.01.

Figure 5S.Forest plot of the 16 statistically significant associations between 1-month milk exosomal miRNA abundance and subsequent infant outcomes. miR-148a shows predominantly negative associations with growth and adiposity; miR-30b shows positive associations at 1 month.

Figure 6S. Heatmap of standardized β coefficients for all miRNA–outcome combinations across 1, 3, and 6-month assessment ages. Asterisks denote p < 0.05 (*), p < 0.01 (**).
