# Supplementary material for: Alterations in Human Milk Adiponectin, Leptin, Resistin, and microRNAs in Mothers With Gestational Diabetes Mellitus Compared With Healthy Controls: A Systematic Review and Network‐Based Analysis
Source: Food Sci Nutr. 2026 Aug 31;14(9):e72256. doi: 10.1002/fsn3.72256 (PMC13528237; doi:10.1002/fsn3.72256)
Supplement: Supplementary file 2 [file FSN3-14-e72256-s002.zip › Supplmentary Files/Bioinformatics/DATA/Data 4.docx]

mRNA Names of Hormones.

| **Hormone/Peptide** | **Gene (mRNA)** |
| --- | --- |
| Leptin | LEP |
| Ghrelin | GHRL |
| Adiponectin | ADIPOQ |
| Resistin | RETN |
| Apelin | APLN |
| Glucagon-like peptide-1 (GLP-1) | GCG |
| Irisin | FNDC5 |
| Nesfatin-1 | NUCB2 |
| Salusin (Salusin-α and Salusin-β) | TOR2A |
| Adropin | ENHO |
| Copeptin | AVP |
