# Supplementary material for: Alterations in Human Milk Adiponectin, Leptin, Resistin, and microRNAs in Mothers With Gestational Diabetes Mellitus Compared With Healthy Controls: A Systematic Review and Network‐Based Analysis
Source: Food Sci Nutr. 2026 Aug 31;14(9):e72256. doi: 10.1002/fsn3.72256 (PMC13528237; doi:10.1002/fsn3.72256)
Supplement: Supplementary file 2 [file FSN3-14-e72256-s002.zip › Supplmentary Files/Systematic Review/Search Strategy.docx]

**The Supplementary File**

Search Strategy

**29 June 2026**

**1. PubMed**

**1.1. GDM, Breast Milk and miRNA**

(((((((mirna[MeSH Terms]) OR (microrna[MeSH Terms])) OR (miRNA[Title/Abstract])) OR (micro RNA[Title/Abstract])) OR (microRNA[Title/Abstract])) OR (miR[Title/Abstract])) AND (((((((diabetes mellitus, gestational[MeSH Terms]) OR (gestational diabetes[MeSH Terms])) OR (gestational diabetes[Title/Abstract])) OR (gestational diabetes mellitus[Title/Abstract])) OR (pregnancy induced diabetes[Title/Abstract])) OR (maternal diabetes[Title/Abstract])) OR (GDM[Title/Abstract]))) AND (((((((((human milk[MeSH Terms]) OR (breast milk[MeSH Terms])) OR (colostrum[MeSH Terms])) OR (human milk[Title/Abstract])) OR (breast milk[Title/Abstract])) OR (human colostrum[Title/Abstract])) OR (breast colostrum[Title/Abstract])) OR (maternal milk[Title/Abstract])) OR (mature milk[Title/Abstract]))

**Results: 8**

**1.2. GDM, Breast Milk and Hormone**

((((((((diabetes mellitus, gestational[MeSH Terms]) OR (gestational diabetes[MeSH Terms])) OR (gestational diabetes[Title/Abstract])) OR (gestational diabetes mellitus[Title/Abstract])) OR (pregnancy induced diabetes[Title/Abstract])) OR (maternal diabetes[Title/Abstract])) OR (GDM[Title/Abstract])) AND (((((((((human milk[MeSH Terms]) OR (breast milk[MeSH Terms])) OR (colostrum[MeSH Terms])) OR (human milk[Title/Abstract])) OR (breast milk[Title/Abstract])) OR (human colostrum[Title/Abstract])) OR (breast colostrum[Title/Abstract])) OR (maternal milk[Title/Abstract])) OR (mature milk[Title/Abstract]))) AND (((((((((((((((((((((obese protein[MeSH Terms]) OR (leptin[MeSH Terms])) OR (ghrelin[MeSH Terms])) OR (adiponectin[MeSH Terms])) OR (resistin[MeSH Terms])) OR (apelin[MeSH Terms])) OR (glucagon like peptide[MeSH Terms])) OR (Obese protein[Title/Abstract])) OR (Ob protein[Title/Abstract])) OR (leptin[Title/Abstract])) OR (ghrelin[Title/Abstract])) OR (adiponectin[Title/Abstract])) OR (resistin[Title/Abstract])) OR (apelin[Title/Abstract])) OR (glucagon like peptide 1[Title/Abstract])) OR (GLP-1[Title/Abstract])) OR (irisin[Title/Abstract])) OR (nesfatin[Title/Abstract])) OR (salusin[Title/Abstract])) OR (adropin[Title/Abstract])) OR (copeptin[Title/Abstract]))

**Results: 25**

**1.3. Combined**

(((((((((diabetes mellitus, gestational[MeSH Terms]) OR (gestational diabetes[MeSH Terms])) OR (gestational diabetes[Title/Abstract])) OR (gestational diabetes mellitus[Title/Abstract])) OR (pregnancy induced diabetes[Title/Abstract])) OR (maternal diabetes[Title/Abstract])) OR (GDM[Title/Abstract])) AND ((((((mirna[MeSH Terms]) OR (microrna[MeSH Terms])) OR (miRNA[Title/Abstract])) OR (micro RNA[Title/Abstract])) OR (microRNA[Title/Abstract])) OR (miR[Title/Abstract]))) AND (((((((((human milk[MeSH Terms]) OR (breast milk[MeSH Terms])) OR (colostrum[MeSH Terms])) OR (human milk[Title/Abstract])) OR (breast milk[Title/Abstract])) OR (human colostrum[Title/Abstract])) OR (breast colostrum[Title/Abstract])) OR (maternal milk[Title/Abstract])) OR (mature milk[Title/Abstract]))) AND (((((((((((((((((((((obese protein[MeSH Terms]) OR (leptin[MeSH Terms])) OR (ghrelin[MeSH Terms])) OR (adiponectin[MeSH Terms])) OR (resistin[MeSH Terms])) OR (apelin[MeSH Terms])) OR (glucagon like peptide[MeSH Terms])) OR (Obese protein[Title/Abstract])) OR (Ob protein[Title/Abstract])) OR (leptin[Title/Abstract])) OR (ghrelin[Title/Abstract])) OR (adiponectin[Title/Abstract])) OR (resistin[Title/Abstract])) OR (apelin[Title/Abstract])) OR (glucagon like peptide 1[Title/Abstract])) OR (GLP-1[Title/Abstract])) OR (irisin[Title/Abstract])) OR (nesfatin[Title/Abstract])) OR (salusin[Title/Abstract])) OR (adropin[Title/Abstract])) OR (copeptin[Title/Abstract]))

**Results: 0**

**2. Scopus**

**2.1. GDM, Breast Milk, and miRNA**

( ( TITLE-ABS-KEY ( breast milk ) OR TITLE-ABS-KEY ( human milk ) OR TITLE-ABS-KEY ( maternal milk ) OR TITLE-ABS-KEY ( human colostrum ) OR TITLE-ABS-KEY ( breast colostrum ) OR TITLE-ABS-KEY ( mature milk ) ) ) AND ( ( TITLE-ABS-KEY ( gestational diabetes ) OR TITLE-ABS-KEY ( diabetes gestational ) OR TITLE-ABS-KEY ( gestational diabetes mellitus ) OR TITLE-ABS-KEY ( pregnancy induced diabetes ) OR TITLE-ABS-KEY ( maternal diabetes ) OR TITLE-ABS-KEY ( GDM ) ) ) AND ( ( TITLE-ABS-KEY ( mirna ) OR TITLE-ABS-KEY ( microrna ) OR TITLE-ABS-KEY ( miR ) OR TITLE-ABS-KEY ( micro RNA ) OR TITLE-ABS-KEY ( mirnas ) ) )

**Results: 27**

**2.2. GDM, Breast Milk, and Hormone**

( ( TITLE-ABS-KEY ( satiety hormone ) OR TITLE-ABS-KEY ( appetite hormone ) OR TITLE-ABS-KEY ( metabolic hormone ) OR TITLE-ABS-KEY ( human milk hormone ) OR TITLE-ABS-KEY ( HM hormone ) OR TITLE-ABS-KEY ( Obese protein ) OR TITLE-ABS-KEY ( Ob protein ) OR TITLE-ABS-KEY ( leptin ) OR TITLE-ABS-KEY ( ghrelin ) OR TITLE-ABS-KEY ( adiponectin ) OR TITLE-ABS-KEY ( irisin ) OR TITLE-ABS-KEY ( salusin ) OR TITLE-ABS-KEY ( nesfatin ) OR TITLE-ABS-KEY ( resistin ) OR TITLE-ABS-KEY ( apelin ) OR TITLE-ABS-KEY ( copeptin ) OR TITLE-ABS-KEY ( adropin ) OR TITLE-ABS-KEY ( glucagon like peptide 1 ) OR TITLE-ABS-KEY ( glp 1 ) ) ) AND ( ( TITLE-ABS-KEY ( breast milk ) OR TITLE-ABS-KEY ( human milk ) OR TITLE-ABS-KEY ( maternal milk ) OR TITLE-ABS-KEY ( human colostrum ) OR TITLE-ABS-KEY ( breast colostrum ) OR TITLE-ABS-KEY ( mature milk ) ) ) AND ( ( TITLE-ABS-KEY ( gestational diabetes ) OR TITLE-ABS-KEY ( diabetes gestational ) OR TITLE-ABS-KEY ( gestational diabetes mellitus ) OR TITLE-ABS-KEY ( pregnancy induced diabetes ) OR TITLE-ABS-KEY ( maternal diabetes ) OR TITLE-ABS-KEY ( GDM ) ) )

**Results: 206**

**2.3. Combined**

( ( TITLE-ABS-KEY ( mirna ) OR TITLE-ABS-KEY ( microrna ) OR TITLE-ABS-KEY ( miR ) OR TITLE-ABS-KEY ( micro RNA ) OR TITLE-ABS-KEY ( mirnas ) ) ) AND ( ( TITLE-ABS-KEY ( gestational diabetes ) OR TITLE-ABS-KEY ( diabetes gestational ) OR TITLE-ABS-KEY ( gestational diabetes mellitus ) OR TITLE-ABS-KEY ( pregnancy induced diabetes ) OR TITLE-ABS-KEY ( maternal diabetes ) OR TITLE-ABS-KEY ( GDM ) ) ) AND ( ( TITLE-ABS-KEY ( breast milk ) OR TITLE-ABS-KEY ( human milk ) OR TITLE-ABS-KEY ( maternal milk ) OR TITLE-ABS-KEY ( human colostrum ) OR TITLE-ABS-KEY ( breast colostrum ) OR TITLE-ABS-KEY ( mature milk ) ) ) AND ( ( TITLE-ABS-KEY ( satiety hormone ) OR TITLE-ABS-KEY ( appetite hormone ) OR TITLE-ABS-KEY ( metabolic hormone ) OR TITLE-ABS-KEY ( human milk hormone ) OR TITLE-ABS-KEY ( HM hormone ) OR TITLE-ABS-KEY ( Obese protein ) OR TITLE-ABS-KEY ( Ob protein ) OR TITLE-ABS-KEY ( leptin ) OR TITLE-ABS-KEY ( ghrelin ) OR TITLE-ABS-KEY ( adiponectin ) OR TITLE-ABS-KEY ( irisin ) OR TITLE-ABS-KEY ( salusin ) OR TITLE-ABS-KEY ( nesfatin ) OR TITLE-ABS-KEY ( resistin ) OR TITLE-ABS-KEY ( apelin ) OR TITLE-ABS-KEY ( copeptin ) OR TITLE-ABS-KEY ( adropin ) OR TITLE-ABS-KEY ( glucagon like peptide 1 ) OR TITLE-ABS-KEY ( glp 1 ) ) )

**Results: 7**

**3. ISI Web of Science**

**3.1. GDM , Breast Milk and miRNA**

**https://www.webofscience.com/wos/woscc/summary/3cd03c61-64a2-4413-ba4f-6bbe42eff493-01bc122be4/relevance/1**

**Results: 12**

**3.2. GDM , Breast Milk and Hormone**

**https://www.webofscience.com/wos/woscc/summary/70b4a4ef-528f-49de-b09e-261d3b870cc1-01bc122811/relevance/1**

**Results: 120**

**3.3. Combined**

**https://www.webofscience.com/wos/woscc/summary/c3c818b7-10d3-4492-9560-f6a85e1bc6b6-01bc122d0f/relevance/1**

**Results: 2**
